# Association of pre- and post-diagnosis dietary total antioxidant capacity (TAC) and composite dietary antioxidant index (CDAI) with overall survival in patients with ovarian cancer: a prospective cohort study

**DOI:** 10.1186/s12967-024-06041-6

**Published:** 2025-01-30

**Authors:** Dong-Dong Wang, Ming-Qian Jia, He-Li Xu, Yu Li, Jia-Xin Liu, Jia-Cheng Liu, Jia-Nan Sun, Fan Cao, Lang Wu, Fang-Hua Liu, Yi-Zi Li, Yi-Fan Wei, Xiao-Ying Li, Qian Xiao, Song Gao, Dong-Hui Huang, Tao Zhang, Ting-Ting Gong, Qi-Jun Wu

**Affiliations:** 1 Department of Clinical Epidemiology, Shengjing Hospital of China Medical University, Shenyang, China; 2 Key Laboratory of Precision Medical Research on Major Chronic Disease, Shengjing Hospital of China Medical University, Shenyang, China; 3 Department of Epidemiology, School of Public Health, China Medical University, Shenyang, China; 4 Department of Obstetrics and Gynecology, Shengjing Hospital of China Medical University, Shenyang, China; 5 Cancer Epidemiology Division, Population Sciences in the Pacific Program, University of Hawaii Cancer Center, University of Hawaii at Manoa, Honolulu, HI USA; 6 Medical Insurance Office, Shengjing Hospital of China Medical University, Shenyang, China; 7 Department of Pediatric, Shengjing Hospital of China Medical University, Shenyang, China; 8 NHC Key Laboratory of Advanced Reproductive Medicine and Fertility (China Medical University), National Health Commission, Shenyang, China

**Keywords:** Cohort study, Composite dietary antioxidant index, Dietary total antioxidant capacity, Ovarian cancer, Survival

## Abstract

**Background:**

The evidence on the relationship of dietary antioxidant nutrients with the survival of ovarian cancer (OC) remains scarce.

**Objective:**

This study aimed to investigate these associations in a prospective cohort of Chinese patients with OC.

**Methods:**

In this prospective cohort study, patients with epithelial OC completed a food frequency questionnaire at diagnosis and 12 months post-diagnosis, and were followed from 2015 to 2023. Dietary total antioxidant capacity (TAC) and composite dietary antioxidant index (CDAI) were calculated based on specific antioxidant nutrients. We examined the associations of pre-diagnosis, post-diagnosis, and changes from pre-diagnosis to post-diagnosis in TAC, CDAI, and representative antioxidant nutrients with overall survival (OS) among patients with OC. Multivariable Cox proportional-hazards models were applied to calculate the hazard ratios (HR) and 95% confidence intervals (CI). Dose–response relationships were evaluated by restricted cubic splines.

**Results:**

Among the total 560 patients with OC, there were 211 (37.68%) deaths during a median follow-up of 44.40 (interquartile range: 26.97–61.37) months. High pre-diagnosis TAC (HR = 0.58; 95% CI 0.38–0.8) and vitamin C intake (HR_T3 vs. T1_ = 0.36; 95% CI 0.21–0.61), and post-diagnosis TAC (HR = 0.57; 95% CI 0.37–0.8), CDAI (HR = 0.57; 95% CI 0.33–0.9), and β-carotene intake (HR_T3 vs. T1_ = 0.55; 95% CI 0.32–0.97) were significantly associated with improved OS. Compared to patients with constantly low pre- and post-diagnosis TAC and CDAI, those with consistently higher TAC (HR_Medium-Medium vs. Low-Low_ = 0.53; 95% CI 0.29–0.97; HR_High-High vs. Low-Low_ = 0.40; 95% CI 0.16–0.94) and CDAI (HR_High-High vs. Low-Low_ = 0.33; 95% CI 0.12–0.88) experienced better OS.

**Conclusion:**

High pre- and post-diagnosis TAC, and post-diagnosis CDAI were associated with improved OC survival, suggesting that consistent high-intake of antioxidant-rich food may be beneficial for the prognosis of OC.

**Supplementary Information:**

The online version contains supplementary material available at 10.1186/s12967-024-06041-6.

## Introduction

Ovarian cancer (OC) stands as a gynecological malignancy characterized by its exceptionally high mortality rate. Based on data from Surveillance, Epidemiology, and End Results Program (1969–2022), the 5-year relative survival for patients with OC is estimated as 50.9% [1]. As a populous country, the burden of OC in China has attracted much attention. A 2022 report indicated that there were approximately 61,100 new cases and 32,600 new deaths attributed to OC in China [2]. Accumulated research illustrated that various lifestyle factors, such as smoking, sub-optional diet, physical inactivity, and alcohol consumption, correlated with worse prognosis in patients with OC [3–5]. Consequently, the alteration of detrimental lifestyle practices emerges as a promising movement to ameliorate survival in OC.

The prognosis of OC is substantially affected by oxidative stress [6]. Overburdens of oxidative stress can lead to DNA damage and gene mutations, thus facilitating the malignant transformation of cells and OC progression [6]. Moreover, oxidative stress can trigger inflammatory responses, and chronic inflammation is known to elevate the risk of OC [7]. Furthermore, oxidative stress may affect the ovarian microenvironment, fostering tumor cell proliferation, invasion, and metastasis [7]. Therefore, it is reasonable to hypothesize that dietary antioxidants may confer protective effects on the prognosis of OC. Previous epidemiological studies have focused on the association of individual antioxidant nutrients or nutrient groups and diverse diseases risk as well as mortality [8–15], but there is a research gap regarding the association between dietary intake of antioxidant nutrients and OC prognosis. Dietary total antioxidant capacity (TAC) and composite dietary antioxidant index (CDAI) are indicators of the cumulative antioxidant capacity of dietary components to scavenge free radicals, and are considered a holistic measure of dietary “total antioxidant content” [16]. However, to the best of our knowledge, there are no studies on the TAC and CDAI in relation to OC survival. Furthermore, current evidence has failed to fully elucidate the association of pre- and post-diagnosis dietary intake of antioxidant nutrients, as well as pre- and post-diagnosis changes in OC survival.

Therefore, this study, based on the Ovarian Cancer Follow-Up Study (OOPS), aims to firstly explore the associations between pre-diagnosis, post-diagnosis, as well as the change from pre-diagnosis to post-diagnosis in TAC and CDAI, with survival in patients with OC.

## Material and method

### Study population

The OOPS is a prospective longitudinal cohort study of patients with OC, aiming to evaluate the relationship between lifestyle factors and health outcomes [17]. Eligible participants were required to meet the following criteria: (1) patients recruited during the baseline survey were between 18 and 79 years old; (2) patients were pathologically diagnosed with epithelial OC; (3) patients’ follow-up and medical treatment were conducted at the gynecological oncology ward at Shengjing Hospital of China Medical University, Shenyang, China after 2015; (4) the operative method was cytoreductive surgery; (5) patients were diagnosed within a 6-month window prior to admission; and (6) patients provided informed consent and demonstrated willingness and capacity to engage in the study and complete the questionnaire.

Participants were excluded if they: (1) receiving chemotherapy at Shengjing Hospital while undergoing surgery at other hospitals; (2) failed to complete or return the administered questionnaire; (3) reported implausible total energy intake (< 500 or > 3,500 kcal/day) in the questionnaire [18]; and (4) declined to participate in this study. The study was granted approval from the Institutional Review Board of the Ethics Committee of Shengjing Hospital of China Medical University (2015PS38K).

### Data collection

In this study, the baseline information was collected through face-to-face, one-to-one interviews by well-trained investigators utilizing self-administered questionnaires [17]. The questionnaire comprehensively encompassed various aspects including general demographic characteristics, dietary supplement usage, sleep pattern, fertility history, dietary history, individual lifestyle, exposure to passive smoking and indoor pollution, physical activity, personal and family medical histories, quality of life, and cognition function. The physical activity levels were quantified using the metabolic equivalent of tasks (MET) from the 2011 update of a comprehensive compendium of physical activities [19], expressed as MET-hour per day (MET·h/d). Anthropometric measurements, including height, weight, waist and hip circumference, and blood pressure, were conducted by trained investigators following a standardized protocol. Body mass index (BMI) was subsequently calculated (BMI = weight in kilograms/height in square meters) using the measured height and weight. Clinical characteristics were abstracted from the electronic medical records of the Shengjing hospital information system, including histologic type (serous and non-serous), degree of pathological differentiation (well, moderate, and poorly differentiated), International Federation of Gynecology and Obstetrics (FIGO) stage (I-II, III-IV, and unknown), presence of residual lesions (yes or no), and comorbidities [20].

### Dietary data collection

Dietary intake data were collected utilizing a 111-item semi-quantitative food frequency questionnaire (FFQ) both at baseline and during follow-up visits by trained investigators. The FFQ has been validated and its reliability has been substantiated in prior studies [21]. The questionnaire categorized the frequency of consumption for most food items across 7 levels (almost never, 2–3 times per month, 1 time per week, 2–3 times per week, 4–6 times per week, 1 time per day, and ≥ 2 times per day). All patients reported the frequencies of each food item consumed in the year preceding their diagnosis and again one year subsequent to their diagnosis. For individual food items, the antioxidant nutrient content was determined by referring to the 2018 Chinese Food Composition Tables [22, 23].

### Estimation of dietary intake of antioxidant nutrients

The theoretical TAC of food items was calculated as the aggregate of products derived from multiplying the antioxidant content by their respective antioxidant capacities [22]. Antioxidant capacities for each antioxidant compound were quantified as vitamin C equivalent antioxidant capacity (VCEAC) values, ascertained vias 2,2’azino-bis-3-ethylbenzthiazoline-6-sulphonic acid assay (ABTS) [22]. The formula was as follows:

$$ TAC = \sum\nolimits_{i = 1}^{19} {(X_{i} *VCEAC_{i} )} $$

The *X*_*i*_ represented the individual antioxidant content (i), and the *VCEAC*_*i*_ represented the VCEAC value (i) of individual antioxidant compounds measured by the ABTS assay. The calculation of TAC incorporated the intake of nineteen antioxidant nutrients including retinol, α-carotene, β-carotene, vitamin C, α-tocopherol, Lutein, Zeaxanthin, Quercetin, Myricetin, Apigenin, Daidzein, Glycitein, Genistein, Delphinidin, Peonidin, Cyanidin, Malvidin, Pelargonidin, and Petundin [24].

The CDAI was calculated using a previously validated formula [25]. The index contained ten minerals and vitamins associated with the antioxidation process and was calculated as the sum of ten nutrients. The formulae were as follows:

$$ CDAI = \sum\nolimits_{i = 1}^{10} {{{(X_{i} - U_{i} )} \mathord{\left/ {\vphantom {{(X_{i} - U_{i} )} {SD_{i} }}} \right. \kern-0pt} {SD_{i} }}} $$

The *X*_*i*_ represented the intake of the CDAI component (i) for each person; the *U*_*i*_ represented the mean of *X*_*i*_ across the cohort for the CDAI component (i), and the *SD*_*i*_ represented the standard deviation of *U*_*i*_ across the cohort. The CDAI components included vitamin A, vitamin C, vitamin E, Magnesium, Selenium, Zinc, Manganese, retinol, α-carotene, and β-carotene [26].

To provide a more granular analysis, individual nutrients known for their antioxidant properties were also quantified as dietary antioxidant index. These included vitamin C, retinol, α-carotene, and β-carotene. By employing these indices and evaluating individual nutrients, we aimed to capture a broad spectrum of dietary antioxidant nutrient intake and its potential impact on OC prognosis.

### Follow-up and ascertainment of outcome

The vital status of OOPS participants was ascertained based on passive and active follow-up mechanisms. In the passive approach, annual follow-up was conducted via a linkage with the Liaoning Providence Center for Disease Control and Prevention every year. Concurrently, clinical specialists gathered patient medical information biannually from the Shengjing Hospital information system, subsequent to the completion of the baseline survey by patients. This ensured that critical information, such as definitive staging, pathology evaluation, diagnosis determination, and initial treatment, was comprehensively recorded. In the active model, patients with OC were followed up through telephone contacts. Survival time was defined as the interval between the date of histologic diagnosis and either death from any cause or the last follow-up (February 16th, 2023) for patients who remained alive. The outcome of interest was overall survival (OS).

### Statistic analysis

The population characteristics were summarized using mean with standard deviations (SDs) or medians [interquartile ranges (IQRs)] for continuous variables and frequencies (percentage) for categorical variables. To assess differences in socio-demographic and clinical characteristics between groups, Student’s t-tests or Kruskal-Wallis tests were applied for continuous variables and the Chi-square tests were employed for categorical variables.

To ensure adequate statistical power, a priori sample size calculations were performed using PASS software [27, 28]. Based on an anticipated coefficient of regression tested by assigning medians of the tertiles for dietary antioxidant nutrient intake in all participants, a significance level of 0.05, and a desired power of 95%, a sample size of 497 participants was estimated.

Crude OS probabilities were estimated and corresponding survival curves were generated through the Kaplan-Meier method [29]. The proportional hazards assumption in Cox models was examined for TAC, CDAI, and individual nutrient intake. For each dietary antioxidant indicator, we tested the assumption against the log-transformed survival time [30]. For the variables that satisfied the proportional hazards assumption, Cox proportional hazards regression models were applied to estimate the adjusted hazard ratios (HRs) and 95% confidence intervals (CIs), quantifying the association of dietary antioxidant nutrient intake before and after diagnosis, as well as changes in dietary antioxidant nutrient intake from pre-diagnosis to post-diagnosis, with OC survival [30]. If the proportional hazards assumption was violated (*P* < 0.05), time-dependent Cox regression models would be adopted instead [31]. The reference category was determined as the lowest tertile of TAC, CDAI, and individual nutrient intake. Trends across tertiles were evaluated by modeling the median value of each tertile as a continuous variable in regression models. TAC, CDAI, and individual nutrient intake was also assessed as a continuous variable per SD increment.

The selection of pertinent covariates was informed by biological plausibility, correlation with exposure, existing literature, and directed acyclic graphs (Supplementary Figure S1) [32]. Covariates included age at diagnosis, age at diagnosis (continuous, years), BMI (< 24 or ≥24 kg/m^2^) [33, 34], and total energy intake (continuous, kcal/day), physical activity (continuous, MET·h/d), income level (< 5,000, 5,000–10,000, or > 10,000 yuan), education level (junior secondary or below, senior high school/technical secondary school, or junior college/university or above), smoking status (yes or no), drinking status (yes or no), tea drinking (yes or no), menopause status (yes or no), use of dietary supplement (yes or no), parity (≤ 1 or ≥ 2), FIGO stage (I-II or III-IV), histological type (serous or non-serous), and residual disease (yes or no). Accordingly, we established three multivariable models for each dietary antioxidant indicator. For model 1, we adjusted for fundamental demographic characteristics. Model 2 expanded upon the first by additionally adjusting for other pertinent baseline characteristics and lifestyle factors. Model 3 incorporated clinical information into an adjustment strategy based on model 2. The detailed information of covariates was presented in Table 2. Restricted cubic spline (RCS) with 3 knots positioned at the 5th, 50th, and 95th percentiles was utilized to dose-response relationships of dietary antioxidant nutrient intake with OC survival [35].

Subgroup analyses were executed, stratified by age at diagnosis (≤ 50 *vs.* > 50 years), BMI (< 24 *vs.* ≥ 24 kg/m^2^), tea (no *vs.* yes), smoking (no *vs.* yes), drinking (no *vs.* yes), FIGO stage (I-II *vs.* III-IV), histological type (serous *vs.* non-serous), menopause status (no *vs.* yes), residual lesions (no *vs.* yes), and parity (≤1 *vs.* ≥2). To further explore potential effect modification, additive and multiplicative interaction analyses were conducted [36]. Interaction terms between the stratifying covariates and dietary antioxidant indicators were incorporated into the Cox regression models. This facilitated an assessment of whether the associations between dietary antioxidant nutrient intake with OC survival differed across subgroups stratified by these demographic and clinical characteristics.

To examine the influence of the change from pre-diagnosis to post-diagnosis in dietary antioxidant nutrient intake on OS, pre-diagnosis and post-diagnosis dietary antioxidant nutrient intake was categorized into low, medium, and high levels by tertiles. Tertile 1 was designated as low, tertile 2 as medium, and tertile 3 as high. This approach enabled the construction of cross-classified change variables, including: low-low (reference), low-medium, low-high, medium-low, medium-medium, medium-high, high-low, high-medium, and high-high. The preceding part and the latter part represented pre-diagnosis and post-diagnosis dietary antioxidant nutrient intake levels, respectively. In addition to categorical change analysis, we also evaluated continuous changes in dietary antioxidant nutrient intake.

To test the robustness of the findings, we not only excluded patients who had a one-year lag before mortality to mitigate reverse causation, but also employed the residual method to adjust dietary antioxidant nutrient intake by performing the regression of total energy intake [37]. Additionally, the E-value methodology was utilized to ascertain the robustness of observed associations between the dietary antioxidant nutrient intake and OC survival, and to estimate the potential impact of unmeasured confounders on the underestimation of associations [38]. All statistical tests were 2-sided, and *P* <0.05 were deemed to represent statistically significant. The analyses were conducted using SAS statistical software (version 9.4; SAS Institute, Cary, NC, USA).

## Result

### Population characteristics

**Fig. 1:**
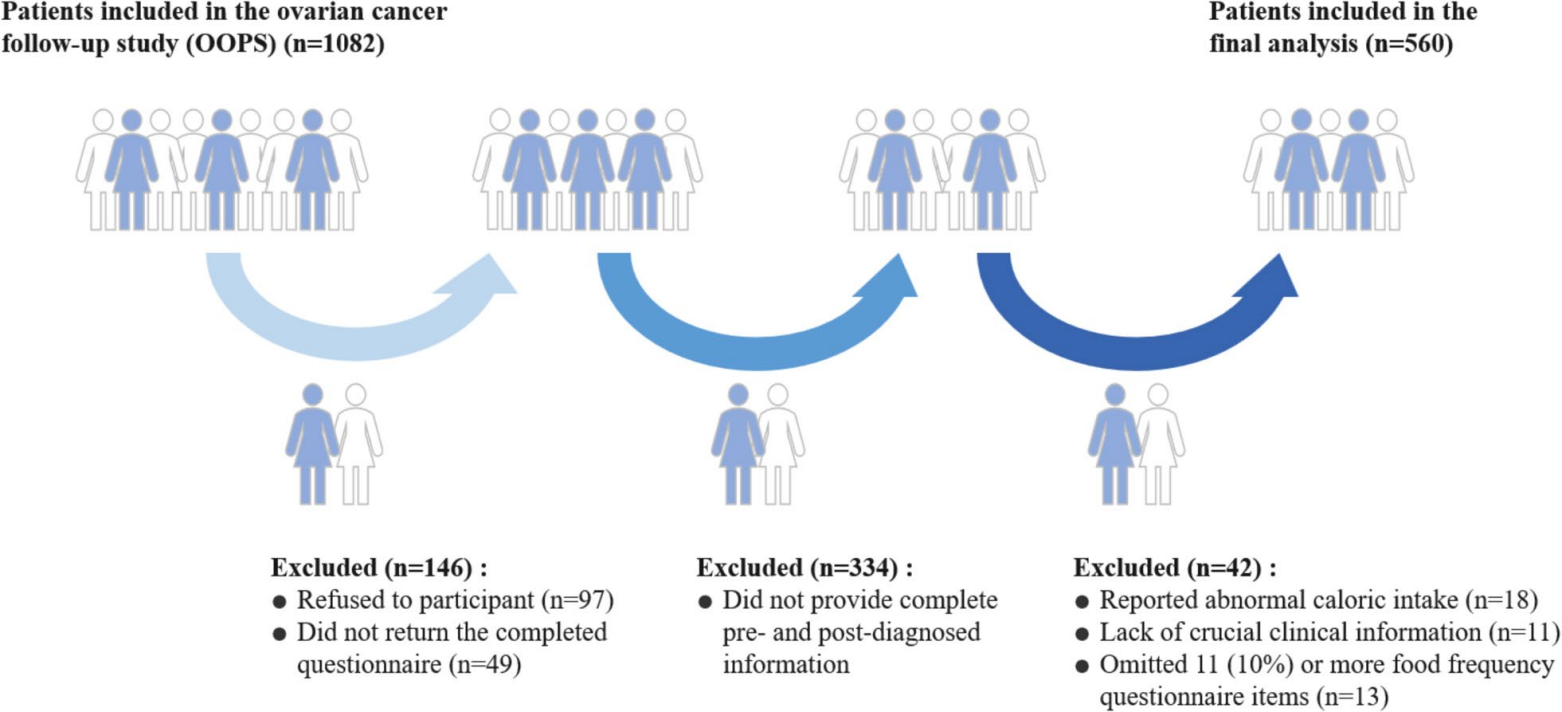
The flow of participants in the Ovarian Cancer Follow-up Study (OOPS)

This study included 560 patients with OC in the final analysis, exceeding the a priori estimated sample size of 497, providing adequate power to detect statistically significant associations. (Figure 1). During the follow-up, we documented 211 (37.68%) total deaths with a median survival time of 44.40 months (IQR: 26.97-61.37). The median age at diagnosis was 53 years, with the majority of cases being III-IV stage (59.11%), serous histology (74.82%), parity ≤ 1 (71.96%), menopausal status (69.64%), and having no residual lesions (79.46%) (Table 1). For cumulative dietary antioxidant nutrient intake, patients with the highest pre-diagnosis TAC and CDAI (in tertile 3) were less likely to be smokers and more likely to use dietary supplements. In addition, patients with the highest pre-diagnosis and post-diagnosis TAC, CDAI, and vitamin C tended to have longer median survival time, a smaller proportion of deaths, and higher total energy intake. Additionally, patients with the highest pre-diagnosis and post-diagnosis TAC were more inclined towards tea consumption (Supplementary Table S1). Kaplan-Meier survival curves were presented in Supplementary Figure S2.

**Table 1:** General characteristics of 560 patients with ovarian cancer grouped by pre- and post-diagnosis

Characteristic	All patients	
	Pre-diagnosis	Post-diagnosis
N (death/total)	211/560	211/560
Survival time (months)	44.40 (26.97–61.37)	44.40 (26.97–61.37)
Age at diagnosis (years)	53.00 (47.00–60.00)	53.00 (47.00–60.00)
Total energy intake (kcal/d)	1347.31 (1046.08–1794.03)	1230.90 (968.97–1624.97)
Physical activity (MET*h/d)	10.76 (5.60–18.40)	6.83 (3.60, 9.00)
BMI (kg/m^2^)		
< 24	360 (64.29)	342 (61.07)
≥ 24	200 (35.71)	218 (38.93)
Smoking status		
No	501 (89.46)	499 (89.11)
Yes	59 (10.54)	61 (10.89)
Alcohol drinking		
No	372 (66.43)	446 (79.64)
Yes	188 (33.57)	114 (20.36)
Tea drinking		
No	308 (55.00)	336 (60.00)
Yes	252 (45.00)	224 (40.00)
Dietary Supplement		
No	449 (80.18)	352 (62.86)
Yes	111 (19.82)	208 (37.14)
Menopause status		
No	170 (30.36)	170 (30.36)
Yes	390 (69.64)	390 (69.64)
Parity		
≤ 1	403 (71.96)	403 (71.96)
≥ 2	157 (28.04)	157 (28.04)
Histological type		
Serous	419 (74.82)	419 (74.82)
Non-serous	141 (25.18)	141 (25.18)
Residual lesions		
No	445 (79.46)	445 (79.46)
Yes	115 (20.54)	115 (20.54)
FIGO stage		
I-II	229 (40.89)	229 (40.89)
III-IV	331 (59.11)	331 (59.11)
Educational level		
Junior secondary or below	328 (58.57)	328 (58.57)
Senior high/ technical secondary school	110 (19.64)	110 (19.64)
Junior college/university or above	122 (21.79)	122 (21.79)
Income per month (yuan)		
< 5000	312 (55.71)	312 (55.71)
5000 to < 10,000	163 (29.11)	163 (29.11)
> 10000	85 (15.18)	85 (15.18)

*BMI* body mass index, *FIGO* The International Federation of Gynecology and Obstetrics, *MET* metabolic equivalents of task

Values are numbers (percentages) for categorical variables and median (interquartile range) for continuous variables

### Pre-diagnosis dietary antioxidant nutrient intake and OC survival

Table 2 revealed the associations between pre-diagnosis cumulative and individual dietary antioxidant nutrient intake and OC survival. In the multivariate model adjusting all potential covariates, for cumulative dietary antioxidant nutrient intake**,** the high pre-diagnosis TAC (HR_T3 vs. T1_ = 0.58; 95% CI 0.38-0.89) was associated with improved OS. For a per-SD increase in pre-diagnosis TAC, the HR and 95% CI for OS was 0.75 (0.62–0.91). For individual dietary antioxidant nutrient intake, the high pre-diagnosis vitamin C (HR_T3 vs. T1_ = 0.36; 95% CI 0.21–0.61) was associated with improved OS. For the per-SD increase in pre-diagnosis vitamin C, the HR and 95% CI for OS was 0.62 (0.48–0.79). Notably, the results of RCS analysis indicated a non-linear relationship between pre-diagnosis α-carotene intake (*P* for nonlinearity < 0.05; *P* for overall association < 0.05) and OS among patients with OC (Supplementary Figure S3).

**Table 2:** Hazard ratio (95% confidence interval) for the associations of pre- and post-diagnosis cumulative and individual dietary antioxidant nutrient intake with overall survival of ovarian cancer

	Pre-diagnosis^3^			*P* for trend^1^	Per-SD increase^2^	Post-diagnosis^4^			*P* for trend^1^	Per-SD increase^2^
	Tertile 1	Tertile 2	Tertile 3			Tertile 1	Tertile 2	Tertile 3		
TAC										
N (death/total)	84/185	66/187	61/188			90/186	61/186	60/188		
Model 1	1.00 (ref.)	0.74 (0.53, 1.04)	0.60 (0.40, 0.92)	0.02	0.78 (0.65, 0.95)	1.00 (ref.)	0.70 (0.49, 0.99)	0.67 (0.44, 1.01)	0.08	0.80 (0.64, 0.99)
Model 2	1.00 (ref.)	0.79 (0.56, 1.11)	0.59 (0.38, 0.90)	0.01	0.78 (0.64, 0.94)	1.00 (ref.)	0.69 (0.49, 0.98)	0.70 (0.46, 1.07)	0.14	0.79 (0.64, 0.99)
Model 3	1.00 (ref.)	0.85 (0.60, 1.20)	0.58 (0.38, 0.89)	0.01	0.75 (0.62, 0.91)	1.00 (ref.)	0.65 (0.46, 0.93)	0.57 (0.37, 0.88)	0.02	0.72 (0.58, 0.90)
CDAI										
N (death/total)	74/186	79/186	58/188			90/186	68/186	53/188		
Model 1	1.00 (ref.)	1.06 (0.74, 1.51)	0.66 (0.38, 1.13)	0.10	0.74 (0.55, 0.98)	1.00 (ref.)	0.76 (0.53, 1.09)	0.51 (0.30, 0.88)	0.02	0.66 (0.48, 0.91)
Model 2	1.00 (ref.)	1.08 (0.75, 1.54)	0.70 (0.41, 1.22)	0.17	0.76 (0.56, 1.03)	1.00 (ref.)	0.81 (0.56, 1.17)	0.56 (0.33, 0.97)	0.04	0.69 (0.50, 0.96)
Model 3	1.00 (ref.)	1.09 (0.76, 1.57)	0.70 (0.40, 1.23)	0.18	0.75 (0.56, 1.02)	1.00 (ref.)	0.76 (0.52, 1.11)	0.57 (0.33, 0.99)	0.05	0.63 (0.45, 0.87)
Vitamin C										
N (death/total)	82/186	74/186	55/188			87/186	67/186	57/188		
Model 1	1.00 (ref.)	0.89 (0.64, 1.25)	0.54 (0.35, 0.83)	0.01	0.76 (0.62, 0.92)	1.00 (ref.)	0.85 (0.61, 1.20)	0.69 (0.45, 1.06)	0.09	0.84 (0.67, 1.05)
Model 2	1.00 (ref.)	0.70 (0.47, 1.05)	0.36 (0.22, 0.62)	0.01	0.64 (0.51, 0.82)	1.00 (ref.)	1.15 (0.71, 1.86)	0.87 (0.48, 1.59)	0.49	0.93 (0.71, 1.21)
Model 3	1.00 (ref.)	0.68 (0.45, 1.03)	0.36 (0.21, 0.61)	0.01	0.62 (0.48, 0.79)	1.00 (ref.)	1.25 (0.77, 2.05)	0.81 (0.44, 1.49)	0.28	0.82 (0.63, 1.08)
Retinol										
N (death/total)	76/186	70/186	65/188			85/186	62/186	64/188		
Model 1	1.00 (ref.)	1.00 (0.70, 1.42)	0.92 (0.64, 1.32)	0.60	1.09 (0.93, 1.28)	1.00 (ref.)	0.71 (0.50, 1.00)	0.72 (0.51, 1.02)	0.14	1.00 (0.85, 1.18)
Model 2	1.00 (ref.)	0.75 (0.51, 1.12)	0.56 (0.34, 0.93)	0.04	1.05 (0.85, 1.29)	1.00 (ref.)	0.82 (0.52, 1.28)	0.73 (0.39, 1.36)	0.38	1.08 (0.87, 1.34)
Model 3	1.00 (ref.)	0.78 (0.53, 1.15)	0.65 (0.39, 1.08)	0.13	1.10 (0.90, 1.34)	1.00 (ref.)	0.88 (0.56, 1.37)	0.89 (0.47, 1.69)	0.79	1.07 (0.86, 1.33)
α-carotene										
N (death/total)	73/186	68/186	70/188			82/186	69/186	60/188		
Model 1	1.00 (ref.)	1.02 (0.73, 1.43)	1.03 (0.72, 1.47)	0.89	0.95 (0.82, 1.11)	1.00 (ref.)	0.90 (0.64, 1.25)	0.78 (0.55, 1.12)	0.19	0.85 (0.72, 1.02)
Model 2	1.00 (ref.)	1.06 (0.72, 1.56)	1.20 (0.76, 1.90)	0.41	1.02 (0.85, 1.21)	1.00 (ref.)	1.00 (0.67, 1.48)	0.97 (0.59, 1.58)	0.88	0.95 (0.76, 1.19)
Model 3	1.00 (ref.)	1.07 (0.72, 1.60)	1.18 (0.74, 1.89)	0.50	0.96 (0.80, 1.14)	1.00 (ref.)	0.97 (0.65, 1.45)	0.95 (0.58, 1.57)	0.86	0.93 (0.74, 1.18)
β-carotene										
N (death/total)	67/186	80/186	64/188			87/186	67/186	57/188		
Model 1	1.00 (ref.)	1.29 (0.92, 1.81)	0.97 (0.66, 1.43)	0.65	0.87 (0.74, 1.01)	1.00 (ref.)	0.73 (0.53, 1.02)	0.67 (0.46, 0.98)	0.05	0.80 (0.66, 0.95)
Model 2	1.00 (ref.)	1.15 (0.77, 1.72)	1.06 (0.64, 1.75)	0.94	0.87 (0.71, 1.08)	1.00 (ref.)	0.64 (0.42, 0.99)	0.61 (0.35, 1.05)	0.12	0.75 (0.58, 0.96)
Model 3	1.00 (ref.)	1.18 (0.77, 1.79)	1.12 (0.66, 1.88)	0.81	0.89 (0.72, 1.10)	1.00 (ref.)	0.56 (0.36, 0.87)	0.55 (0.32, 0.97)	0.08	0.71 (0.55, 0.92)

All HR (95% CI) were calculated using Cox proportional hazard models

*CDAI* composite dietary antioxidant index, *Ref* reference, *SD* standard deviation, *TAC* total antioxidant capacity

^1^*P* for trend was tested by assigning medians of the tertiles for cumulative and individual dietary antioxidant nutrient intake in all patients

^2^Percentage change in ovarian cancer survival for each standard deviation increase in cumulative and individual dietary antioxidant nutrient intake

^3^Pre-diagnosis models: TAC: Model 1 adjusted for age at diagnosis, pre-diagnosis body mass index (BMI), pre-diagnosis total energy intake; Model 2 same as model 1 and further adjusted for pre-diagnosis physical activity, income, education, pre-diagnosis smoking status, pre-diagnosis alcohol drinking, pre-diagnosis tea drinking, pre-diagnosis dietary supplement, parity, menopause status; Model 3 same as model 2 and further adjusted for histological type, residual lesions, and the International Federation of Gynecology and Obstetrics stage. CDAI: Model 1 adjusted for age at diagnosis, pre-diagnosis body mass index (BMI), pre-diagnosis total energy intake; Model 2 same as model 1 and further adjusted for pre-diagnosis physical activity, income, education, pre-diagnosis smoking status, pre-diagnosis alcohol drinking, pre-diagnosis dietary supplement, parity, menopause status; Model 3 same as model 2 and further adjusted for histological type, residual lesions, and the International Federation of Gynecology and Obstetrics stage. Vitamin C, Retinol, α-carotene,and β-carotene: Model 1 adjusted for age at diagnosis, pre-diagnosis body mass index (BMI), pre-diagnosis total energy intake; Model 2 same as model 1 and further adjusted for pre-diagnosis physical activity, income, education, pre-diagnosis smoking status, pre-diagnosis alcohol drinking, pre-diagnosis dietary supplement, parity, menopause status, and other dietary antioxidant nutrient intake before diagnosis; Model 3 same as model 2 and further adjusted for histological type, residual lesions, and the International Federation of Gynecology and Obstetrics stage

^4^Post-diagnosis models: TAC: Model 1 adjusted for age at diagnosis, post-diagnosis BMI, post-diagnosis total energy intake; Model 2 same as model 1 and further adjusted for post-diagnosis physical activity, income, education, post-diagnosis smoking status, post-diagnosis alcohol drinking, post-diagnosis tea drinking, post-diagnosis dietary supplement, menopause status, parity; Model 3 same as model 2 and further adjusted for histological type, residual lesions, and the International Federation of Gynecology and Obstetrics stage. CDAI: Model 1 adjusted for age at diagnosis, post-diagnosis BMI, post-diagnosis total energy intake; Model 2 same as model 1 and further adjusted for post-diagnosis physical activity, income, education, post-diagnosis smoking status, post-diagnosis alcohol drinking, post-diagnosis dietary supplement, menopause status, parity; Model 3 same as model 2 and further adjusted for histological type, residual lesions, and the International Federation of Gynecology and Obstetrics stage. Vitamin C, Retinol, α-carotene,and β-carotene: Model 1 adjusted for age at diagnosis, post-diagnosis BMI, post-diagnosis total energy intake; Model 2 same as model 1 and further adjusted for post-diagnosis physical activity, income, education, post-diagnosis smoking status, post-diagnosis alcohol drinking, post-diagnosis dietary supplement, menopause status, parity, and other dietary antioxidant nutrient intake after diagnosis; Model 3 same as model 2 and further adjusted for histological type, residual lesions, and the International Federation of Gynecology and Obstetrics stage

### Post-diagnosis dietary antioxidant nutrient intake and OC survival

As shown in Table 2, when comparing the top tertile to the bottom tertile, for cumulative dietary antioxidant nutrient intake, higher post-diagnosis TAC (HR_T3 vs. T1_ = 0.57; 95% CI 0.37-0.88) and CDAI (HR_T3 vs. T1_ = 0.57; 95% CI 0.33-0.99) tended to be associated with better OS of OC. For a per-SD increase in post-diagnosis TAC and CDAI, the HR for OC were 0.72 (0.58–0.90) and 0.63 (0.45–0.87), respectively. For individual dietary antioxidant nutrient intake, higher post-diagnosis β-carotene (HR_T3 vs. T1_ = 0.55; 95% CI 0.32–0.97) tended to be associated with better OS of OC. For a per-SD increase in post-diagnosis β-carotene, the HR and 95%CI for OS was 0.71 (0.55–0.92). In contrast, the pre-diagnosis vitamin C, retinol, and α-carotene did not exhibit significant correlations with OS in patients with OC. The RCS analysis indicated no significant non-linear association between any dietary antioxidant nutrients index and OC survival (Supplementary Figure S3).

### Changes in dietary antioxidant nutrient intake with OC survival

We investigated the association of changes in dietary antioxidant nutrient intake from pre-diagnosis to post-diagnosis with OS among patients with OC. For cumulative dietary antioxidant nutrient intake, compared to patients with persistently low levels of TAC both before and after diagnosis (Low-Low), those who consistently maintained higher levels of TAC experienced more favorable OS outcomes (HR_Medium-Medium vs. Low-Low_ = 0.53; 95% CI 0.29–0.97; HR_High-High vs. Low-Low_ = 0.40; 95% CI 0.16–0.94) (Supplementary Table S7). Similarly, patients with consistently high levels (measured as the categorical levels) CDAI had a survival advantage (HR_High-High vs. Low-Low_ = 0.33; 95% CI 0.12–0.88). (Supplementary Table S7). Notably, a reduction in TAC by more than 30% (measured as the continuous absolute difference) from pre-diagnosis to post-diagnosis, led to a 92% increase in mortality risk (95% CI 1.27–2.89) (Table 3). Correspondingly, 30% and more decrease in CDAI yielded an 81% increase in mortality risk (95% CI 1.18–2.77) (Table 4). For individual dietary antioxidant nutrient intake, a reduction in retinol intake exceeding 0.4 (measured as the continuous absolute difference) was associated with a 146% increase in mortality risk (95% CI 1.42–4.24). Similarly, a reduction greater than 0.4 in β-carotene linked to a 91% increase in mortality risk (95% CI 1.10–3.31) (Table 5).

**Table 3:** Associations between changes in pre- and post-diagnosis total antioxidant capacity and overall survival of ovarian cancer

	Decrease (> 30%)	No change or stable (± ≤ 30%)	Increase (> 30%)
N (death/total)	56/115	87/266	68/179
Model 1	2.13 (1.47, 3.07)	1.00 (ref.)	0.94 (0.65, 1.34)
Model 2	1.68 (1.13, 2.51)	1.00 (ref.)	0.82 (0.55, 1.23)
Model 3	1.92 (1.27, 2.89)	1.00 (ref.)	0.79 (0.52, 1.20)

Ref, reference

All hazard ratios (95% confidence intervals) were calculated using Cox proportional hazard models

Model 1 adjusted for age at diagnosis, pre-diagnosis body mass index (BMI), pre-diagnosis total energy intake, pre-diagnosis physical activity, income, education, pre-diagnosis smoking status, pre-diagnosis alcohol drinking, pre-diagnosis tea drinking, pre-diagnosis dietary supplement, menopause status, parity, and pre-diagnosis total antioxidant capacity

Model 2 same as model 1 and further adjusted for change in BMI, change in total energy intake, change in physical activity, change in smoking status, change in alcohol drinking, change in tea drinking, and change in dietary supplement

Model 3 same as model 2 and further adjusted for histological type, residual lesions, and the International Federation of Gynecology and Obstetrics stage

**Table 4:** Associations between changes in pre- and post-diagnosis composite dietary antioxidant index and overall survival of ovarian cancer

	Decrease (> 3)	No change or stable (± ≤ 3)	Increase (> 3)
N (death/total)	82/168	82/236	47/156
Model 1	2.13 (1.51, 3.02)	1.00 (ref.)	0.85 (0.59, 1.24)
Model 2	1.56 (1.03, 2.38)	1.00 (ref.)	0.73 (0.48, 1.11)
Model 3	1.81 (1.18, 2.77)	1.00 (ref.)	0.72 (0.47, 1.10)

Ref, reference

All hazard ratios (95% confidence intervals) were calculated using Cox proportional hazard models

Model 1 adjusted for age at diagnosis, pre-diagnosis body mass index (BMI), pre-diagnosis total energy intake, pre-diagnosis physical activity, income, education, pre-diagnosis smoking status, pre-diagnosis alcohol drinking, pre-diagnosis dietary supplement, menopause status, parity, and pre-diagnosis composite dietary antioxidant index

Model 2 same as model 1 and further adjusted for change in BMI, change in total energy intake, change in physical activity, change in smoking status, change in alcohol drinking, and change in dietary supplement

Model 3 same as model 2 and further adjusted for histological type, residual lesions, and the International Federation of Gynecology and Obstetrics stage

**Table 5:** Associations between changes in pre- and post-diagnosis individual dietary antioxidant nutrient intake and overall survival of ovarian cancer

	Decrease (> 0.4)	No change or stable (± ≤ 0.4)	Increase (> 0.4)
Vitamin C			
N (death/total)	68/157	88/256	55/147
Model 1	1.42 (0.90, 2.22)	1.00 (ref.)	0.88 (0.59, 1.30)
Model 2	0.94 (0.54, 1.64)	1.00 (ref.)	0.70 (0.39, 1.28)
Model 3	1.06 (0.60, 1.90)	1.00 (ref.)	0.95 (0.52, 1.76)
Retinol			
N (death/total)	56/115	103/311	52/134
Model 1	2.14 (1.37, 3.34)	1.00 (ref.)	1.16 (0.79, 1.71)
Model 2	1.96 (1.09, 3.54)	1.00 (ref.)	1.48 (0.77, 2.85)
Model 3	2.13 (1.17, 3.87)	1.00 (ref.)	1.22 (0.64, 2.31)
α-carotene			
N (death/total)	54/121	113/311	44/128
Model 1	1.56 (0.95, 2.56)	1.00 (ref.)	0.82 (0.55, 1.23)
Model 2	1.36 (0.72, 2.57)	1.00 (ref.)	0.97 (0.56, 1.67)
Model 3	1.61 (0.84, 3.09)	1.00 (ref.)	0.88 (0.49, 1.57)
β-carotene			
N (death/total)	66/152	100/267	45/141
Model 1	1.27 (0.82, 1.98)	1.00 (ref.)	0.67 (0.44, 1.02)
Model 2	0.95 (0.53, 1.70)	1.00 (ref.)	0.41 (0.22, 0.77)
Model 3	1.40 (0.77, 2.57)	1.00 (ref.)	0.47 (0.25, 0.87)

Ref, reference

All hazard ratios (95% confidence intervals) were calculated using Cox proportional hazard models

Model 1 adjusted for age at diagnosis, pre-diagnosis body mass index (BMI), pre-diagnosis total energy intake, pre-diagnosis physical activity, income, education, pre-diagnosis smoking status, pre-diagnosis alcohol drinking, pre-diagnosis dietary supplement, menopause status, parity, and pre-diagnosis individual dietary antioxidant nutrient intake

Model 2 same as model 1 and further adjusted for change in BMI, change in total energy intake, change in physical activity, change in smoking status, change in alcohol drinking, change in dietary supplement, and change in other dietary antioxidant nutrient intake

Model 3 same as model 2 and further adjusted for histological type, residual lesions, and the International Federation of Gynecology and Obstetrics stage

### Subgroup and interaction analyses

In the subgroup analyses, for cumulative dietary antioxidant nutrient intake, a significant multiplicative interaction was discerned in relation to age at diagnosis and pre-diagnosis CDAI. Besides, significant additive interactions were presented between smoking and pre-diagnosis TAC, between alcohol drinking and post-diagnosis TAC, as well as between alcohol drinking and post-diagnosis CDAI, all of which were statistically significant (all *P* < 0.05) (Supplementary Table S8).

For individual dietary antioxidant nutrient intake, significant multiplicative interactions were detected between smoking status and pre-diagnosis α-carotene and β-carotene intake, as well as between age at diagnosis and pre-diagnosis retinol intake (all *P* < 0.05) (Supplementary Table S9). Notable additive interactions were identified, including between smoking status and pre-diagnosis intake levels of vitamin C, α-carotene, and β-carotene, between alcohol drinking and post-diagnosis vitamin C, and between age at diagnosis and post-diagnosis retinol (all *P* < 0.05) (Supplementary Table S9).

### Sensitivity analysis

The sensitivity analysis of pre-diagnosis dietary antioxidant nutrient intake yielded results that aligned with the primary findings after excluding patients with a one-year lag before mortality and modulating for total energy intake utilizing the residual method (Supplementary Table S10). All the E-values can be considered relatively large, indicating that the results were robust (Supplementary Table S11).

For post-diagnosis dietary antioxidant nutrient intake, the sensitivity analysis excluding patients with a one-year lag before mortality showed consistent results for vitamin C, retinol, α-carotene, and β-carotene. However, findings for post-diagnosis TAC and CDAI were altered. After adjusting total energy intake using the residual method, findings for TAC, CDAI, vitamin C, retinol, and α-carotene remained unaltered, whereas the finding for β-carotene was affected (Supplementary Table S10). All the E-values were relatively substantial, indicating that the results were robust (Supplementary Table S11).

## Discussion

This investigation is, to our knowledge, the inaugural study to evaluate the relationship between dietary antioxidant nutrient intake with OS of patients with OC within a Chinese demographic. Our prospective cohort study revealed that both high pre-diagnosis and post-diagnosis TAC were associated with improved OS of OC. Moreover, high post-diagnosis CDAI was similarly associated with favorable OC survival. When examining changes from pre-diagnosis to post-diagnosis in TAC and CDAI, the consistently high TAC and CDAI were associated with better OS when compared to those with persistently low TAC or CDAI.

TAC and CDAI have been reported to be associated with a heightened risk of all-cause mortality in multiple types of cancer and chronic disease [39–43]. The US National Health and Nutrition Examination Survey, for instance, indicated an inverse relationship between TAC and non-cancer mortality in cancer survivors [44]. It was noteworthy that prior studies examining the association between TAC and the risk of OC yielded no significant findings [45, 46]. Additionally, despite TAC representing an index of accumulative antioxidant capacity, several studies have concentrated on the significance of specific individual antioxidant nutrients for OC prognosis [47–49]. For instance, Sun et al. reported that pre-diagnosis higher consumption of certain antioxidant vitamins, including vitamin C and β-carotene, was related to more favorable survival outcomes in OC according to the prior OOPS study [11]. Our analysis of identical nutrients shared the same findings with them. CDAI was a scoring system used to assess the overall antioxidant content of an individual's diet and to indicate the body's antioxidant capacity [25]. Previous studies have found that CDAI was inversely correlated with common markers of oxidative stress and inflammation, such as IL-1β and TNF-α levels [26]. Wang et al. demonstrated that high CDAI levels were associated with a reduced risk of all-cause and cardiovascular mortality [41].

Subgroup analyses revealed nuanced associations between TAC and CDAI with OS. The beneficial association of TAC with survival was pronounced among non-tea drinkers, whereas the results in the tea-drinker subgroup failed to reach statistical significance. This observation could be attributed to the potent antioxidant activity of tea polyphenols [50], which may overlap with those from an antioxidant-rich diet. For individuals already consuming tea, the antioxidants derived from tea polyphenols might be adequate in mitigating oxidative stress, rendering the incremental benefits of TAC relatively negligible. Notably, for non-drinking patients, both pre- and post-diagnostic TAC and CDAI were significantly associated with improved OC survival. These findings could suggest that in the absence of alcohol, antioxidants may play a more pronounced role in influencing patient survival. This observation aligns with previous research demonstrating that alcohol consumption can impair antioxidant absorption and utilization [51], and induce oxidative stress through multiple pathways [52]. Moreover, alcohol has been shown to interfere with the body's natural antioxidant defense mechanisms and may counteract the beneficial effects of dietary antioxidants [51]. Furthermore, pre-diagnosis TAC was associated with improved survival in patients with non-serous OC, whereas post-diagnosis TAC was significantly linked to prolonged OS in those diagnosed with serous OC. This suggested that the reliance on oxidant stress signaling pathways may vary, influencing the impact of TAC on survival outcomes [6]. For patients with advanced stage (FIGO stage III-IV) OC, the progression of the tumor and metabolic abnormalities have already led to severe oxidative stress in the body [53–55]. In this situation, pre-diagnosis TAC may not fully represent the actual antioxidative capacity. After diagnosis, the comprehensive treatments (surgery, chemotherapy, etc.) received by advanced-stage patients may alter the body's oxidative stress status [56, 57], potentially allowing TAC to influence prognosis more significantly.

Our findings revealed that alcohol drinking can modify the association of post-diagnosis TAC and CDAI with the survival of patients with OC. The observed additive interactions could hold significance for public health initiatives by aiding in the identification of patients with OC who might derive greater benefit from dietary modifications to increase antioxidant-rich food intake. For instance, neither pre-diagnosis nor post-diagnosis TAC and CDAI were significantly associated with improved survival in OC among patients who consumed alcohol. There was also an additive interaction between smoking status and pre-diagnosis TAC. Both smoking and alcohol consumption are primary contributors to exogenous oxidative stress, resulting in persistent oxidative damage in the body [58, 59]. Such a high level of oxidative stress may overwhelm the antioxidant capacity provided by an antioxidant-rich diet, diminishing the potential benefits of antioxidants on survival.

Interestingly, when we examined changes from pre-diagnosis to post-diagnosis in TAC in relation to survival, OC survivors who consistently maintained higher TAC exhibited better survival compared to those who persistently had low TAC. In contrast, a transition from low to high TAC did not lead to significant enhancements in survival. This phenomenon suggested that consistently high levels of TAC, rather than improvements in intake, are crucial for improved survival. These findings highlighted the importance of maintaining a consistently high dietary antioxidant capacity in enhancing survival outcomes.

This study had several notable strengths. At the forefront, it was the inaugural study to explore the association of pre-diagnosis and post-diagnosis TAC and CDAI with OC survival within the Chinese population. The prospective design and high response rate employed in this study minimized the potential for recall bias [60]. Secondly, the collection of patient-related clinical information and lifestyle factors enabled the meticulous statistical adjustment for potential confounders. Importantly, our study also included post-diagnosis TAC and CDAI data. Finally, we conducted extensive subgroup analyses as well as examined the interactions between TAC as well as CDAI and various demographic and clinical factors, thereby improving the reliability of the findings.

Nonetheless, our study also had some limitations. Firstly, the reliance on FFQ to collect dietary information the year before diagnosis and one year after diagnosis may introduce exposure misclassification and recall bias [61]. To compensate for this, we arranged for face-to-face interviews to be conducted by trained nurses. Secondly, OOPS is a single-center prospective cohort study, the generalizability of the results may be limited [62]. Thirdly, the relatively small sample size of our cohort diminished the statistical power for subgroup analyses, which did not allow us to rule out the possibility of chance finding. This underscores the need for future multi-center studies with large sample sizes. Fourthly, despite the comprehensive statistical adjustments, the possibility of residual confounding by unmeasured factors, such as mutation types and specific clinical treatments [63–67], remains a concern. Nevertheless, the estimated E-values for OC survival within our study were considered relatively higher than the acknowledged staple risk factors of OC mortality [65–68], i.e. wild-type BRCA mutation. This suggested that the evidence for the observed positive association of pre-diagnosis and post-diagnosis TAC and CDAI with OC survival was robust to unmeasured confounders. Finally, our study focused solely on assessing the OS of OC since OC-specific survival was not available. However, epidemiological evidence suggested that the total mortality may be analogous to the disease-specific mortality in patients with OC due to the typically poor prognosis, where most patients with this malignancy ultimately succumb to the cancer itself [1, 69].

## Conclusion

Our study supports a positive association between both pre-diagnosis and post-diagnosis dietary antioxidant intake and improved OS among patients with OC. Consistently high levels of TAC were significantly associated with better survival, suggesting the importance of maintaining an antioxidant-rich diet. This finding could have important clinical implications, potentially guiding dietary recommendations for patients with OC and individuals at risk. Additionally, it should be noted that lifestyle health factors, such as smoking and alcohol consumption, may counteract the beneficial impact of dietary antioxidant intake. To substantiate these findings, additional prospective studies of large scale and extended duration are required.

## Supplementary Information

Additional file 1.

## Data Availability

The data that support the findings of our study are available from the corresponding author upon reasonable request.

## Abbreviations

BMI: Body mass index
CDAI: Composite dietary antioxidant index
CIs: Confidence intervals
TAC: Dietary total antioxidant capacity
FIGO: Federation of Gynecology and Obstetrics
FFQ: Food frequency questionnaire
HRs: Hazard ratios
IQRs: Interquartile ranges
MET: Metabolic equivalent of tasks
OC: Ovarian cancer
OOPS: Ovarian Cancer Follow-Up Study
OS: Overall survival
RCS: Restricted cubic spline
SDs: Standard deviations
VCEAC: Vitamin C equivalent antioxidant capacity
