# Supplementary material for: Association of pre- and post-diagnosis dietary total antioxidant capacity (TAC) and composite dietary antioxidant index (CDAI) with overall survival in patients with ovarian cancer: a prospective cohort study
Source: J Transl Med. 2025 Jan 30;23:134. doi: 10.1186/s12967-024-06041-6 (PMC11783755; doi:10.1186/s12967-024-06041-6)
Supplement: Supplementary file 1 — Additional file 1. [file 12967_2024_6041_MOESM1_ESM.docx]

**Supplementary Materials**

**Supplementary Figure S1**


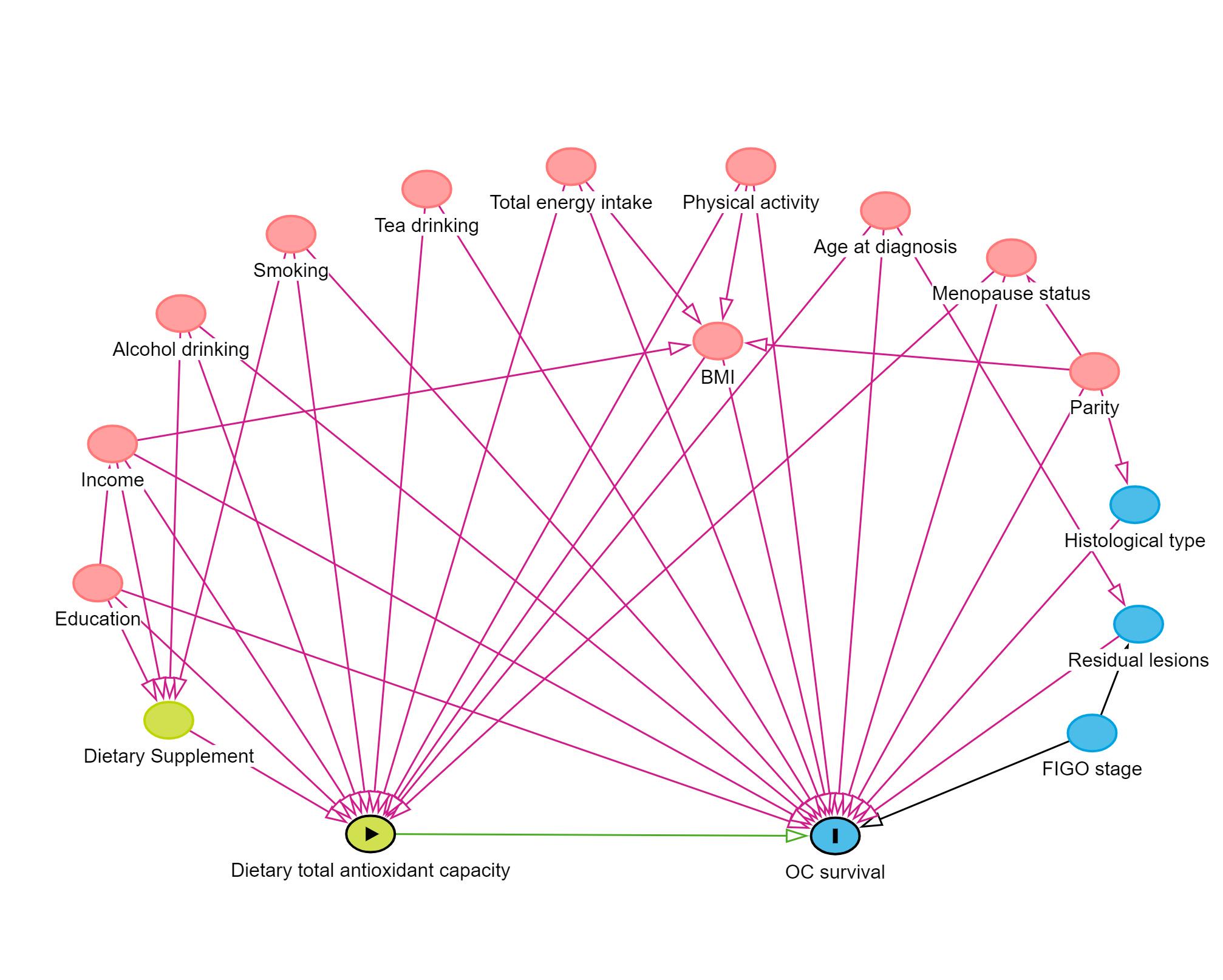

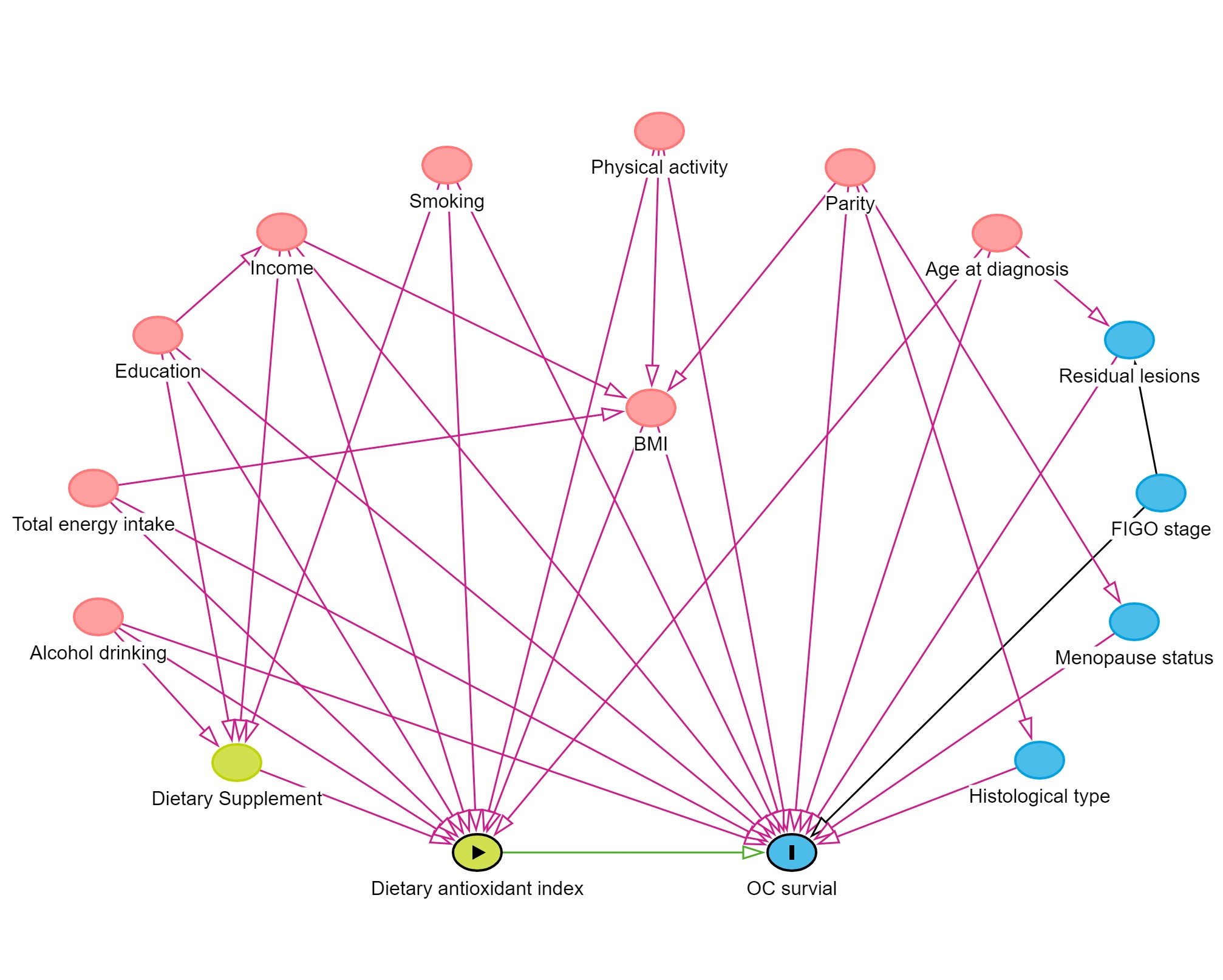


**Supplementary Figure S1: Directed Acyclic Graph of the associations of total antioxidant capacity and dietary antioxidant index with overall survival of ovarian cancer**

BMI, body mass index; FIGO, The International Federation of Gynecology and Obstetrics; OC, ovarian cancer.

The directed acyclic graph represents associations between covariates and primary exposure and outcome. Pink circles represent ancestors of exposure and outcome (i.e., confounders), green circles represent ancestors of the exposure (i.e., causal determinants of the exposure), blue circles represent ancestors of the outcome (i.e., causal determinants of the outcome). Green lines represent causal paths, and pink lines represent biasing paths. The minimally sufficient adjustment set represents covariates such that the adjustment for this set of variables will minimize confounding bias when estimating the association between the exposure and the outcome. The minimally sufficient adjustment set was determined using the DAGitty website (https://www.dagitty.net/dags.html). The final minimally sufficient adjustment set for total antioxidant capacity comprised age at diagnosis, body mass index, alcohol drinking, education, income, menopause status, physical activity, smoking, tea drinking, total energy intake. The final minimally sufficient adjustment set for dietary antioxidant index comprised age at diagnosis, alcohol drinking, BMI, education, income, physical activity, smoking, total energy intake.

**References**

1. Goodman MT, Tung KH. Alcohol consumption and the risk of borderline and invasive ovarian cancer. Obstet Gynecol. 2003;101(6):1221-8. doi: 10.1016/s0029-7844(03)00050-4.

2. Fortner RT, Trewin-Nybraten CB, Paulsen T, Langseth H. Characterization of ovarian cancer survival by histotype and stage: A nationwide study in Norway. Int J Cancer. 2023;153(5):969-78. doi: 10.1002/ijc.34576.

3. Pan SY, DesMeules M. Energy intake, physical activity, energy balance, and cancer: epidemiologic evidence. Methods Mol Biol. 2009;472:191-215. doi: 10.1007/978-1-60327-492-0_8.

4. Altman AD, Lambert P, Love AJ, Turner D, Lotocki R, Dean E, Popowich S, Nachtigal MW. Examining the Effects of Time to Diagnosis, Income, Symptoms, and Incidental Detection on Overall Survival in Epithelial Ovarian Cancer: Manitoba Ovarian Cancer Outcomes (MOCO) Study Group. Int J Gynecol Cancer. 2017;27(8):1637-44. doi: 10.1097/IGC.0000000000001074.

5. Hansen JM, Nagle CM, Ibiebele TI, Grant PT, Obermair A, Friedlander ML, DeFazio A, Webb PM, Ovarian Cancer P, Lifestyle Study G. A healthy lifestyle and survival among women with ovarian cancer. Int J Cancer. 2020;147(12):3361-9. doi: 10.1002/ijc.33155.

6. Bae HS, Kim HJ, Hong JH, Lee JK, Lee NW, Song JY. Obesity and epithelial ovarian cancer survival: a systematic review and meta-analysis. J Ovarian Res. 2014;7:41. doi: 10.1186/1757-2215-7-41.

7. Yancik R. Ovarian cancer. Age contrasts in incidence, histology, disease stage at diagnosis, and mortality. Cancer. 1993;71(2 Suppl):517-23. doi: 10.1002/cncr.2820710205.

8. Jones TL, Sandler CX, Spence RR, Hayes SC. Physical activity and exercise in women with ovarian cancer: A systematic review. Gynecol Oncol. 2020;158(3):803-11. doi: 10.1016/j.ygyno.2020.06.485.

9. Weiderpass E, Oh JK, Algeri S, Bellocco R. Socioeconomic status and epithelial ovarian cancer survival in Sweden. Cancer Causes Control. 2014;25(8):1063-73. doi: 10.1007/s10552-014-0407-1.

10. Matz M, Coleman MP, Carreira H, Salmeron D, Chirlaque MD, Allemani C, Group CW. Worldwide comparison of ovarian cancer survival: Histological group and stage at diagnosis (CONCORD-2). Gynecol Oncol. 2017;144(2):396-404. doi: 10.1016/j.ygyno.2016.11.019.

11. van der Burg ME. Advanced ovarian cancer. Curr Treat Options Oncol. 2001;2(2):109-18. doi: 10.1007/s11864-001-0053-1.

12. L'Esperance K, Grundy A, Abrahamowicz M, Arseneau J, Gilbert L, Gotlieb WH, Provencher D, Koushik A. Alcohol intake and the risk of epithelial ovarian cancer. Cancer Causes Control. 2023;34(6):533-41. doi: 10.1007/s10552-023-01681-3.

13. Dunneram Y, Greenwood DC, Cade JE. Diet, menopause and the risk of ovarian, endometrial and breast cancer. Proc Nutr Soc. 2019;78(3):438-48. doi: 10.1017/S0029665118002884.

14. Asghari G, Yuzbashian E, Shahemi S, Gaeini Z, Mirmiran P, Azizi F. Dietary total antioxidant capacity and incidence of chronic kidney disease in subjects with dysglycemia: Tehran Lipid and Glucose Study. Eur J Nutr. 2018;57(7):2377-85. doi: 10.1007/s00394-017-1511-2.

15. Kim K, Vance TM, Chen MH, Chun OK. Dietary total antioxidant capacity is inversely associated with all-cause and cardiovascular disease death of US adults. Eur J Nutr. 2018;57(7):2469-76. doi: 10.1007/s00394-017-1519-7.

16. Carvalho MR, Crivellenti LC, Sartorelli DS. Estimate of Dietary Total Antioxidant Capacity of Pregnant Women and Associated Factors. Rev Bras Ginecol Obstet. 2022;44(2):91-9. doi: 10.1055/s-0041-1741454.

17. Jun S, Chun OK, Joung H. Estimation of total antioxidant capacity of Korean adults. Eur J Nutr. 2018;57(4):1615-25. doi: 10.1007/s00394-017-1447-6.

18. Zhang M, Lee AH, Binns CW, Xie X. Green tea consumption enhances survival of epithelial ovarian cancer. Int J Cancer. 2004;112(3):465-9. doi: 10.1002/ijc.20456.

19. Rooth C. Ovarian cancer: risk factors, treatment and management. Br J Nurs. 2013;22(17):S23-30. doi: 10.12968/bjon.2013.22.Sup17.S23.

20. Okubo H, Shaheen SO, Ntani G, Jameson KA, Syddall HE, Sayer AA, Dennison EM, Cooper C, Robinson SM, Hertfordshire Cohort Study G. Processed meat consumption and lung function: modification by antioxidants and smoking. Eur Respir J. 2014;43(4):972-82. doi: 10.1183/09031936.00109513.

21. Sasanfar B, Toorang F, Maleki F, Esmaillzadeh A, Zendehdel K. Association between total antioxidant capacity and breast cancer: a case-control study in a Middle Eastern country. Public Health Nutr. 2021;24(5):965-72. doi: 10.1017/S1368980019004397.

22. Mozaffari H, Daneshzad E, Larijani B, Surkan PJ, Azadbakht L. Association of total antioxidant capacity to anthropometry in healthy women: A cross-sectional study. Nutrition. 2020;69:110577. doi: 10.1016/j.nut.2019.110577.

23. Langton CR, Whitcomb BW, Purdue-Smithe AC, Sievert LL, Hankinson SE, Manson JE, Rosner BA, Bertone-Johnson ER. Association of Parity and Breastfeeding With Risk of Early Natural Menopause. JAMA Netw Open. 2020;3(1):e1919615. doi: 10.1001/jamanetworkopen.2019.19615.

24. Pellegrini N, Salvatore S, Valtuena S, Bedogni G, Porrini M, Pala V, et al. Development and validation of a food frequency questionnaire for the assessment of total antioxidant capacity. J Nutr. 2007;137(1):93-8. doi: 10.1093/jn/137.1.93.

25. Henriquez-Sanchez P, Sanchez-Villegas A, Ruano-Rodriguez C, Gea A, Lamuela-Raventos RM, Estruch R, et al. Dietary total antioxidant capacity and mortality in the PREDIMED study. Eur J Nutr. 2016;55(1):227-36. doi: 10.1007/s00394-015-0840-2.

26. Gaitskell K, Green J, Pirie K, Barnes I, Hermon C, Reeves GK, Beral V, Million Women Study C. Histological subtypes of ovarian cancer associated with parity and breastfeeding in the prospective Million Women Study. Int J Cancer. 2018;142(2):281-9. doi: 10.1002/ijc.31063.

27. Voguet L, Hebert T, Leveque J, Acker O, Mesbah H, Marret H, Poree P, Body G. Patient age and positive margins are predictive factors of residual tumor on mastectomy specimen after conservative treatment for breast cancer. Breast. 2009;18(4):233-7. doi: 10.1016/j.breast.2009.06.002.

28. Costanzo S, De Curtis A, di Niro V, Olivieri M, Morena M, De Filippo CM, et al. Postoperative atrial fibrillation and total dietary antioxidant capacity in patients undergoing cardiac surgery: The Polyphemus Observational Study. J Thorac Cardiovasc Surg. 2015;149(4):1175-82 e1. doi: 10.1016/j.jtcvs.2014.11.035.

29. Mao J, Zhao Y, Hu H, Zhou M, Yang X. An L-shaped association between composite dietary antioxidant index and stroke: Evidence from NHANES 2011-2020. J Stroke Cerebrovasc Dis. 2024;33(3):107578. doi: 10.1016/j.jstrokecerebrovasdis.2024.107578.

30. O'Brien SK, Malacova E, Sherriff JL, Black LJ. The Prevalence and Predictors of Dietary Supplement Use in the Australian Population. Nutrients. 2017;9(10). doi: 10.3390/nu9101154.

31. Sheng LT, Jiang YW, Pan A, Koh WP. Dietary total antioxidant capacity and mortality outcomes: the Singapore Chinese Health Study. Eur J Nutr. 2022;61(5):2375-82. doi: 10.1007/s00394-022-02812-3.

32. Song S, Youn J, Lee YJ, Kang M, Hyun T, Song Y, Lee JE. Dietary supplement use among cancer survivors and the general population: a nation-wide cross-sectional study. BMC Cancer. 2017;17(1):891. doi: 10.1186/s12885-017-3885-1.

33. Tan ECK, Eshetie TC, Gray SL, Marcum ZA. Dietary Supplement Use in Middle-aged and Older Adults. J Nutr Health Aging. 2022;26(2):133-8. doi: 10.1007/s12603-022-1732-9.

34. Vahid F, Rahmani W, Khodabakhshi A, Davoodi SH. Associated between Dietary Antioxidant Index (DAI) and Odds of Breast Cancer and Correlation between DAI with Pathobiological Markers: Hospital-Based Incidence Case-Control Study. J Am Nutr Assoc. 2023;42(4):386-92. doi: 10.1080/07315724.2022.2056543.

35. Wang H, Chen Y. Relationship between Composite Dietary Antioxidant Index and Aging. Healthcare (Basel). 2023;11(20). doi: 10.3390/healthcare11202722.

36. Wang T, Liu H, Wei X. Association between the Composite Dietary Antioxidant Index and Stroke: A cross-sectional Study. Biol Trace Elem Res. 2023. doi: 10.1007/s12011-023-04011-5.

37. Wang Z, Li WT, Gao Y, Xue X, Pang H, Hao W, et al. Analysis of Dietary Supplement Use and Influencing Factors in the Mongolian Population. Biomed Res Int. 2022;2022:4064588. doi: 10.1155/2022/4064588.

38. Yu YC, Paragomi P, Wang R, Jin A, Schoen RE, Sheng LT, Pan A, Koh WP, Yuan JM, Luu HN. Composite dietary antioxidant index and the risk of colorectal cancer: Findings from the Singapore Chinese Health Study. Int J Cancer. 2022;150(10):1599-608. doi: 10.1002/ijc.33925.

39. Wang W, Wang X, Cao S, Duan Y, Xu C, Gan D, He W. Dietary Antioxidant Indices in Relation to All-Cause and Cause-Specific Mortality Among Adults With Diabetes: A Prospective Cohort Study. Front Nutr. 2022;9:849727. doi: 10.3389/fnut.2022.849727.

40. Zhao L, Sun Y, Cao R, Wu X, Huang T, Peng W. Non-linear association between composite dietary antioxidant index and depression. Front Public Health. 2022;10:988727. doi: 10.3389/fpubh.2022.988727.

41. Zhao L, Zhang X, Guo S, Han K, Sun Y, Li X, Yan Z, WeiPeng. Relationship between composite dietary antioxidant index and depression among overweight and obese adults. J Affect Disord. 2023;341:358-65. doi: 10.1016/j.jad.2023.08.140.

**Supplementary Figure S2**


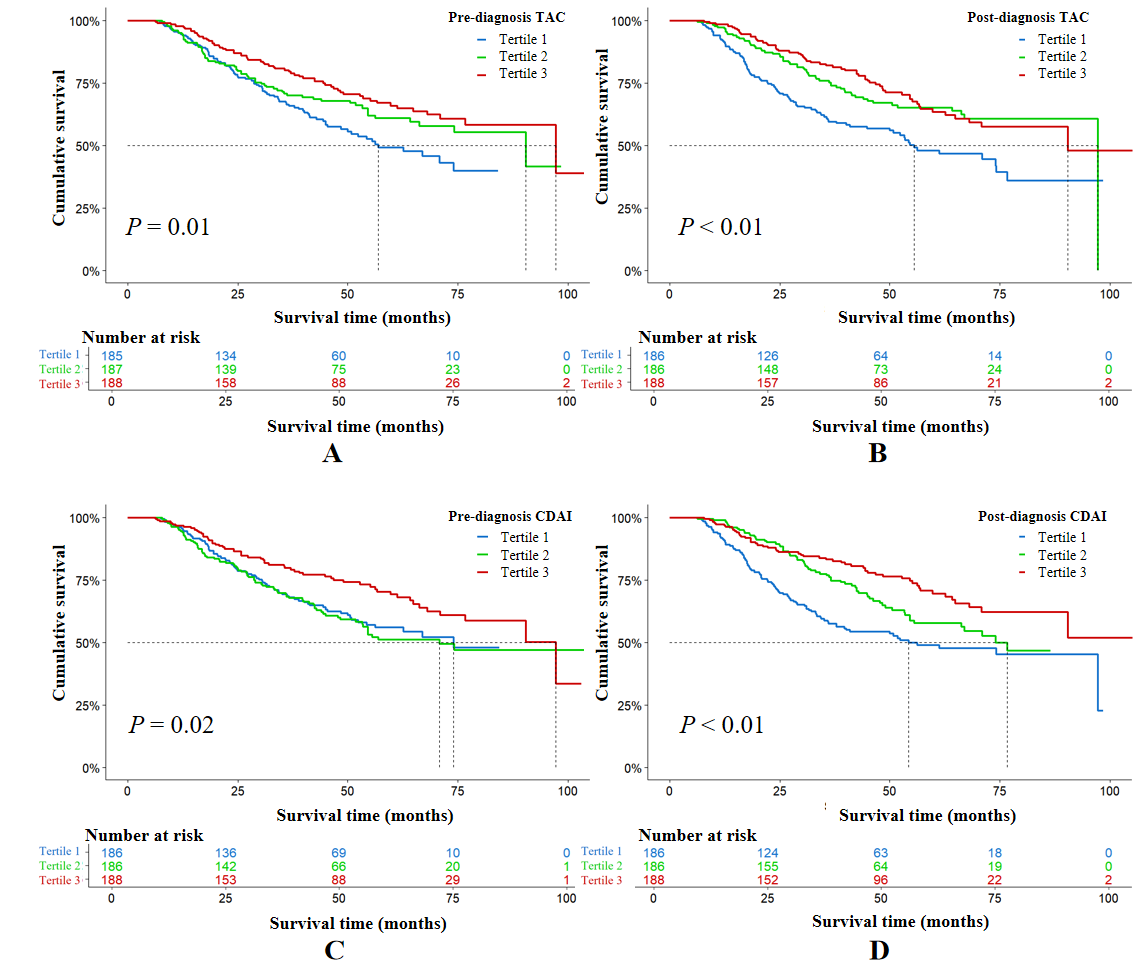

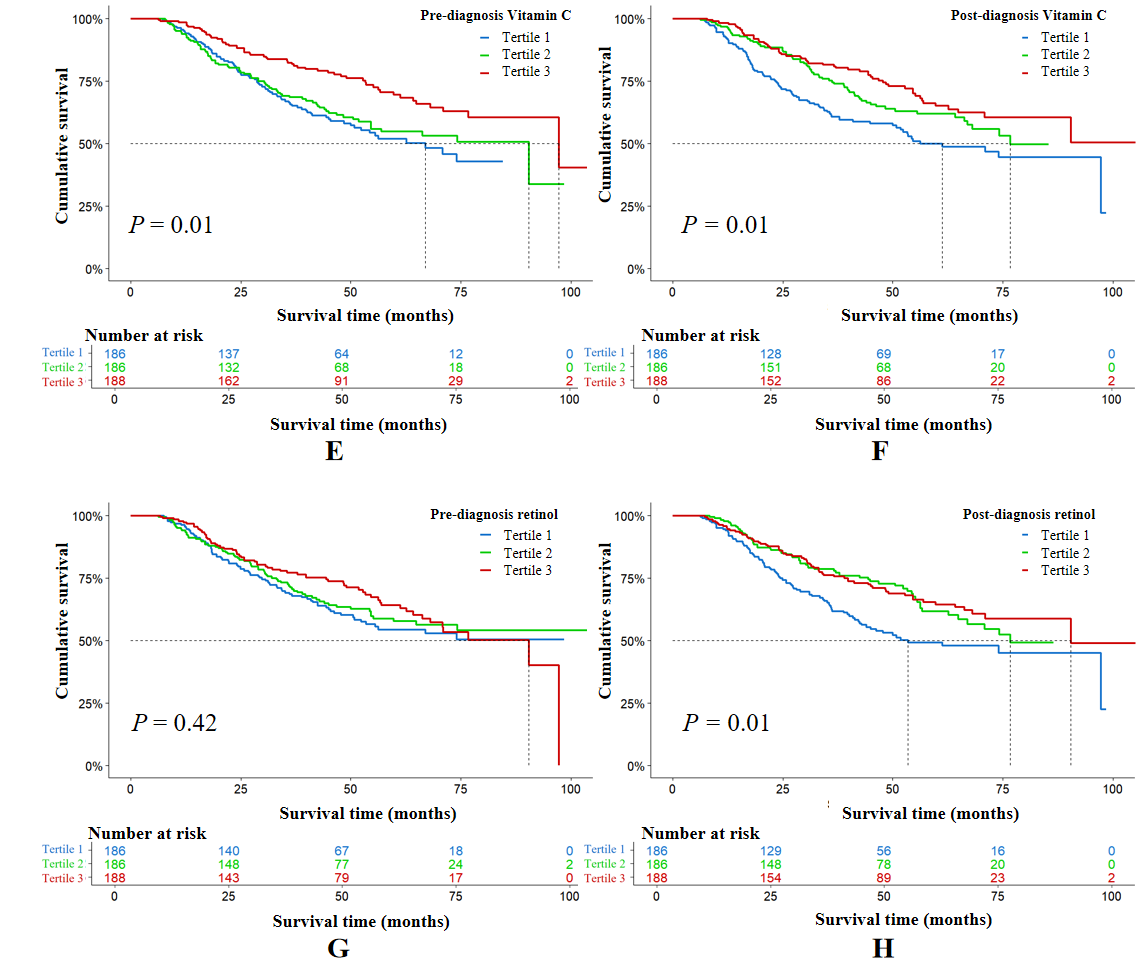

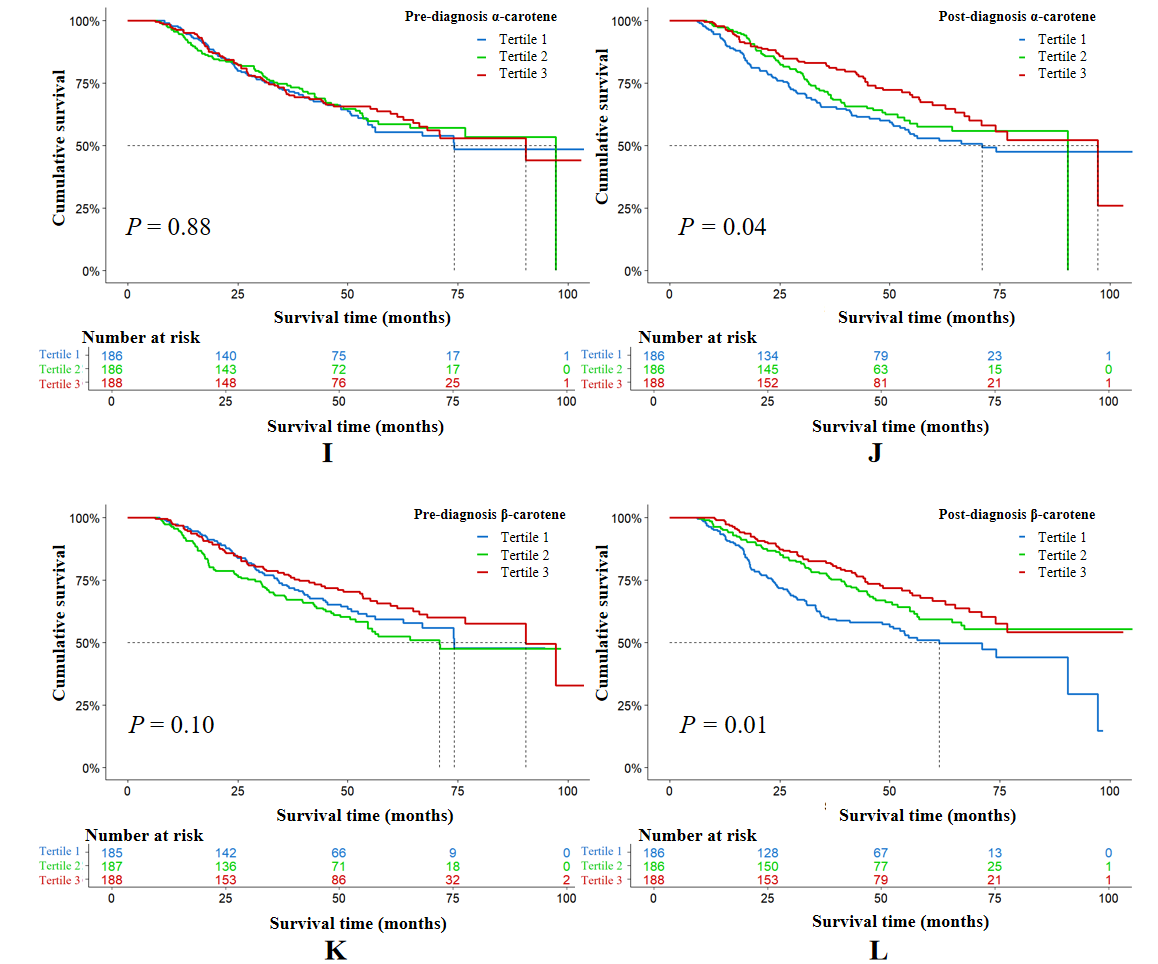


**Supplementary Figure S2: Kaplan–Meier survival curves for (A) pre-diagnosis total antioxidant capacity (TAC), (B) post-diagnosis TAC, (C) pre-diagnosis composite dietary antioxidant index (CDAI), (D) post-diagnosis CDAI, (E) pre-diagnosis Vitamin C, (F) post-diagnosis Vitamin C, (G) pre-diagnosis retinol, (H) post-diagnosis retinol, (I) pre-diagnosis α-carotene, (J) post-diagnosis α-carotene, (K) pre-diagnosis β-carotene, (L) and post-diagnosis β-carotene**

**Supplementary Figure S3**


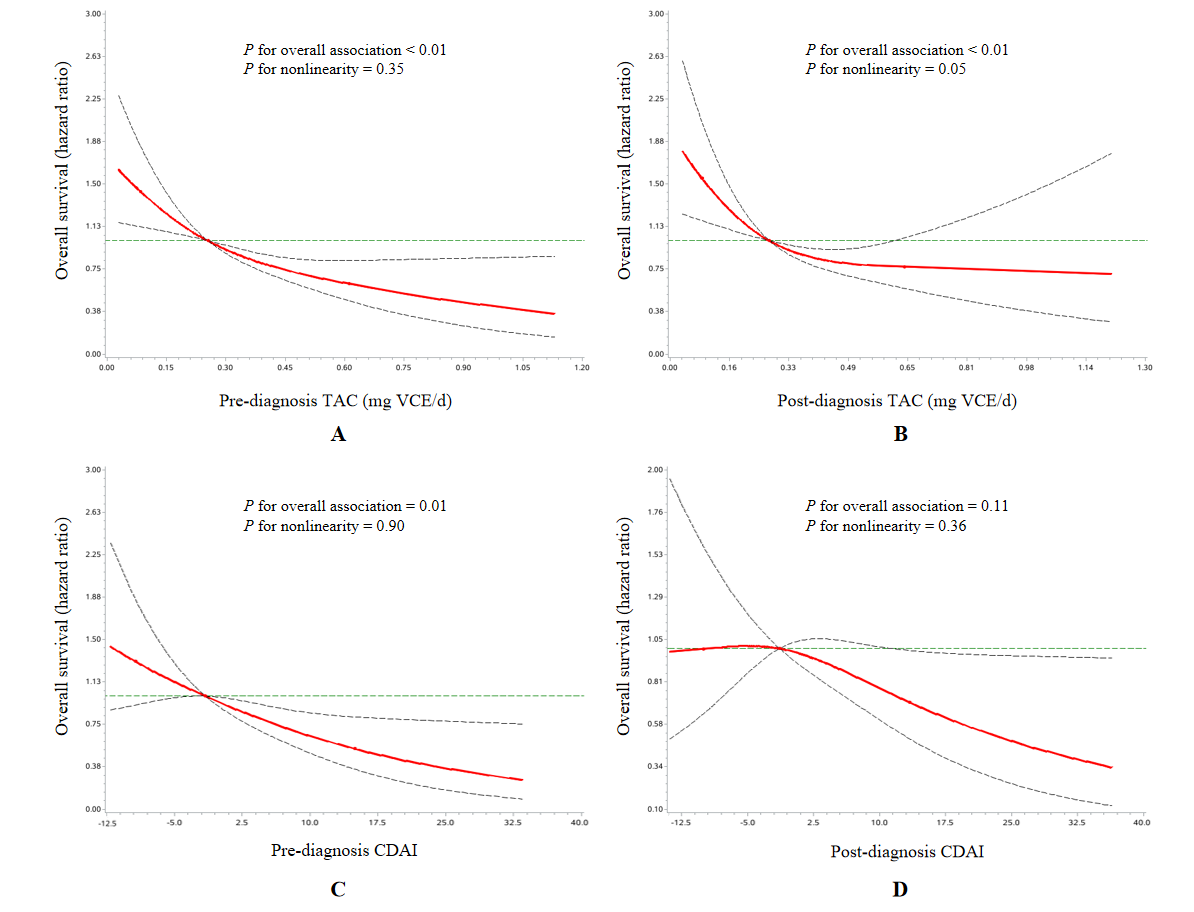

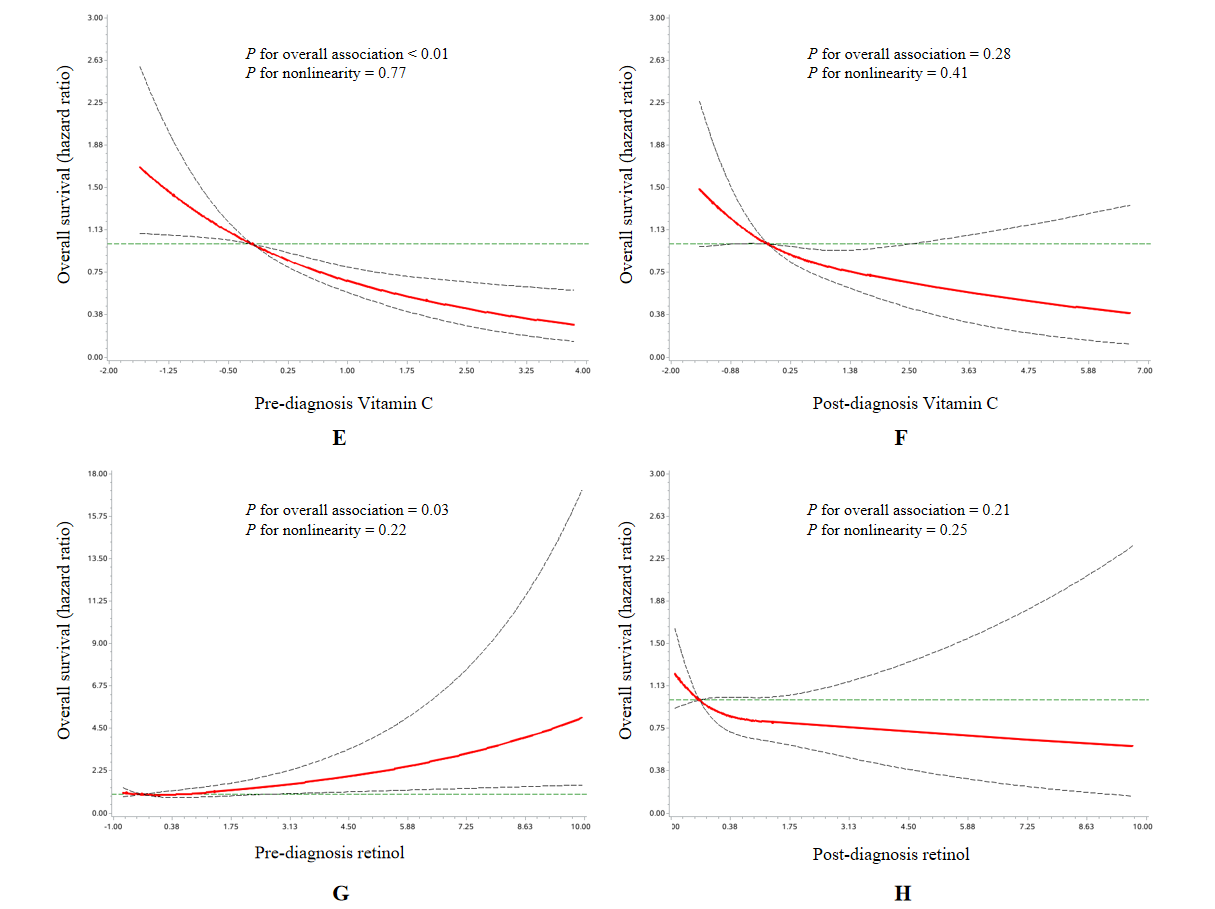

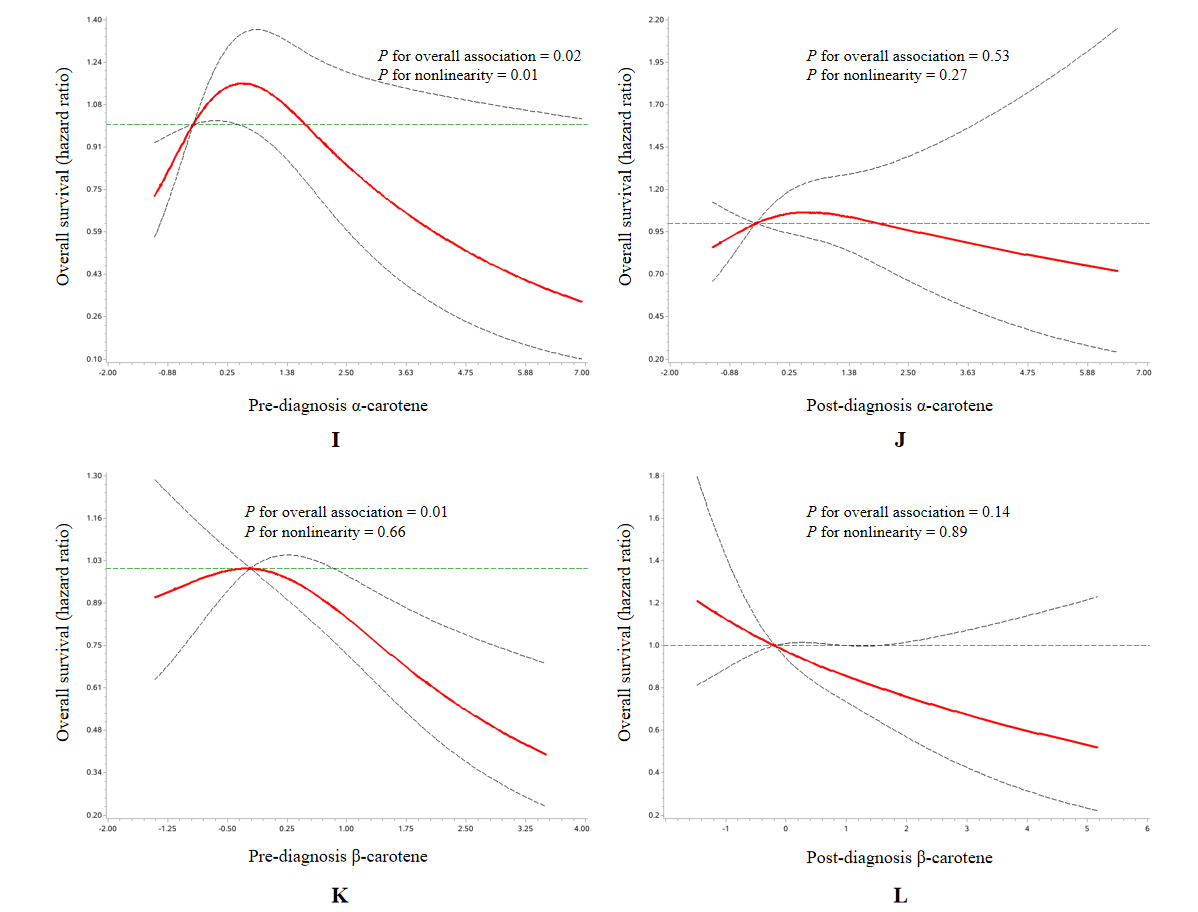


**Supplementary Figure S3: Visualization of the relationship between overall survival of ovarian cancer and (A)** **pre-diagnosis total antioxidant capacity (TAC), (B) post-diagnosis TAC, (C) pre-diagnosis composite dietary antioxidant index (CDAI)****, (D) post-diagnosis CDAI, (E) pre-diagnosis Vitamin C, (F) post-diagnosis Vitamin C, (G) pre-diagnosis retinol, (H) post-diagnosis retinol, (I) pre-diagnosis α-carotene, (J) post-diagnosis α-carotene, (K) pre-diagnosis β-carotene, (L) and post-diagnosis β-carotene using restricted cubic splines analyses**

Solid lines are multivariable adjusted hazard ratios, with dash lines showing 95% confidence intervals derived from restricted cubic splines analyses with three knots.

Pre-diagnosis TAC model was adjusted for pre-diagnosis body mass index (BMI), pre-diagnosis total energy intake, age at diagnosis, pre-diagnosis physical activity, income, education, pre-diagnosis smoking status, pre-diagnosis alcohol drinking, pre-diagnosis tea drinking, pre-diagnosis dietary supplement, menopause status, parity, histological type, residual lesions, and the International Federation of Gynecology and Obstetrics stage.

Post-diagnosis TAC model was adjusted for post-diagnosis BMI, post-diagnosis total energy intake, age at diagnosis, post-diagnosis physical activity, income, education, post-diagnosis smoking status, post-diagnosis alcohol drinking, post-diagnosis tea drinking, post-diagnosis dietary supplement, menopause status, parity, histological type, residual lesions, and the International Federation of Gynecology and Obstetrics stage.

Pre-diagnosis CDAI model was adjusted for pre-diagnosis body mass index (BMI), pre-diagnosis total energy intake, age at diagnosis, pre-diagnosis physical activity, income, education, pre-diagnosis smoking status, pre-diagnosis alcohol drinking, pre-diagnosis dietary supplement, menopause status, parity, histological type, residual lesions, and the International Federation of Gynecology and Obstetrics stage.

Post-diagnosis CDAI model was adjusted for post-diagnosis BMI, post-diagnosis total energy intake, age at diagnosis, post-diagnosis physical activity, income, education, post-diagnosis smoking status, post-diagnosis alcohol drinking, post-diagnosis dietary supplement, menopause status, parity, histological type, residual lesions, and the International Federation of Gynecology and Obstetrics stage.

Pre-diagnosis individual dietary antioxidant nutrients intake model was adjusted for pre-diagnosis body mass index (BMI), pre-diagnosis total energy intake, age at diagnosis, pre-diagnosis physical activity, income, education, pre-diagnosis smoking status, pre-diagnosis alcohol drinking, pre-diagnosis dietary supplement, menopause status, parity, histological type, residual lesions, the International Federation of Gynecology and Obstetrics stage, and pre-diagnosis DAI of other nutrient.

Post-diagnosis individual dietary antioxidant nutrients intake model model was adjusted for post-diagnosis BMI, post-diagnosis total energy intake, age at diagnosis, post-diagnosis physical activity, income, education, post-diagnosis smoking status, post-diagnosis alcohol drinking, post-diagnosis dietary supplement, menopause status, parity, histological type, residual lesions, the International Federation of Gynecology and Obstetrics stage, and post-diagnosis DAI of other nutrient.

**Supplementary Table S1. General characteristics of 560 patients with ovarian cancer grouped by tertiles of pre-diagnosis and post-diagnosis** **total antioxidant capacity**

| **Characteristic** | **Pre-diagnosis TAC** | | | ***P* value** | **Post-diagnosis TAC** | | | ***P* value** |
| --- | --- | --- | --- | --- | --- | --- | --- | --- |
|  | **Tertile 1** | **Tertile 2** | **Tertile 3** |  | **Tertile 1** | **Tertile 2** | **Tertile 3** |  |
| **N (death/total)** | 84/185 | 66/187 | 61/188 | 0.03 | 90/186 | 61/186 | 60/188 | 0.01 |
| **Survival time (months)** | 40.93 (23.57-55.67) | 44.00 (24.63-62.40) | 48.20 (32.40-68.07) | 0.01 | 37.32 (20.30-55.67) | 43.85 (28.53-64.57) | 48.27 (31.32-63.43) | 0.01 |
| **Age at diagnosis (years)** | 53.00 (47.00-58.00) | 54.00 (48.00-59.00) | 53.00 (47.00-61.50) | 0.69 | 54.00 (49.00-60.00) | 52.00 (47.00-59.00) | 53.00 (47.00-60.50) | 0.35 |
| **Total energy intake (kcal/d)** | 1021.94 (848.34-1262.45) | 1318.33 (1111.87-1683.31) | 1795.52 (1465.61-2199.12) | 0.01 | 957.56 (744.56-1101.39) | 1240.69 (1028.69-1543.94) | 1627.83 (1369.00-1869.85) | 0.01 |
| **Physical activity (MET*h/d)** | 11.70 (6.10-17.80) | 9.81 (5.80-16.20) | 9.81 (5.16-20.79) | 0.57 | 5.40 (3.60-9.00) | 6.83 (3.60-9.00) | 7.20 (3.60-9.00) | 0.33 |
| **BMI (kg/m^2^)** |  |  |  | 0.11 |  |  |  | 0.15 |
| < 24 | 120 (64.86) | 110 (58.82) | 130 (69.15) |  | 124 (66.67) | 107 (57.53) | 111 (59.04) |  |
| ≥ 24 | 65 (35.14) | 77 (41.18) | 58 (30.85) |  | 62 (33.33) | 79 (42.47) | 77 (40.96) |  |
| **Smoking status** |  |  |  | 0.02 |  |  |  | 0.69 |
| No | 156 (84.32) | 173 (92.51) | 172 (91.49) |  | 163 (87.63) | 166 (89.25) | 170 (90.43) |  |
| Yes | 29 (15.68) | 14 (7.49) | 16 (8.51) |  | 23 (12.37) | 20 (10.75) | 18 (9.57) |  |
| **Alcohol drinking** |  |  |  | 0.17 |  |  |  | 0.64 |
| No | 131 (70.81) | 125 (66.84) | 116 (61.70) |  | 151 (81.18) | 144 (77.42) | 151 (80.32) |  |
| Yes | 54 (29.19) | 62 (33.16) | 72 (38.30) |  | 35 (18.82) | 42 (22.58) | 37 (19.68) |  |
| **Tea drinking** |  |  |  | 0.02 |  |  |  | 0.01 |
| No | 116 (62.70) | 102 (54.55) | 90 (47.87) |  | 125 (67.20) | 113 (60.75) | 98 (52.13) |  |
| Yes | 69 (37.30) | 85 (45.45) | 98 (52.13) |  | 61 (32.80) | 73 (39.25) | 90 (47.87) |  |
| **Dietary Supplement** |  |  |  | 0.05 |  |  |  | 0.01 |
| No | 154 (83.24) | 155 (82.89) | 140 (74.47) |  | 131 (70.43) | 119 (63.98) | 102 (54.26) |  |
| Yes | 31 (16.76) | 32 (17.11) | 48 (25.53) |  | 55 (29.57) | 67 (36.02) | 86 (45.74) |  |
| **Menopause status** |  |  |  | 0.09 |  |  |  | 0.68 |
| No | 64 (34.59) | 60 (32.09) | 46 (24.47) |  | 58 (31.18) | 52 (27.96) | 60 (31.91) |  |
| Yes | 121 (65.41) | 127 (67.91) | 142 (75.53) |  | 128 (68.82) | 134 (72.04) | 128 (68.09) |  |
| **Parity** |  |  |  | 0.85 |  |  |  | 0.21 |
| ≤ 1 | 136 (73.51) | 133 (71.12) | 134 (71.28) |  | 131 (70.43) | 128 (68.82) | 144 (76.60) |  |
| ≥ 2 | 49 (26.49) | 54 (28.88) | 54 (28.72) |  | 55 (29.57) | 58 (31.18) | 44 (23.40) |  |
| **Histological type** |  |  |  | 0.39 |  |  |  | 0.68 |
| Serous | 137 (74.05) | 135 (72.19) | 147 (78.19) |  | 142 (76.34) | 135 (72.58) | 142 (75.53) |  |
| Non-serous | 48 (25.95) | 52 (27.81) | 41 (21.81) |  | 44 (23.66) | 51 (27.42) | 46 (24.47) |  |
| **Residual lesions** |  |  |  | 0.31 |  |  |  | 0.13 |
| No | 148 (80.00) | 154 (82.35) | 143 (76.06) |  | 149 (80.11) | 155 (83.33) | 141 (75.00) |  |
| Yes | 37 (20.00) | 33 (17.65) | 45 (23.94) |  | 37 (19.89) | 31 (16.67) | 47 (25.00) |  |
| **FIGO stage** |  |  |  | 0.85 |  |  |  | 0.59 |
| I-II | 74 (40.00) | 75 (40.11) | 80 (42.55) |  | 76 (40.86) | 81 (43.55) | 72 (38.30) |  |
| III-IV | 111 (60.00) | 112 (59.89) | 108 (57.45) |  | 110 (59.14) | 105 (56.45) | 116 (61.70) |  |
| **Educational level** |  |  |  | 0.01 |  |  |  | 0.23 |
| Junior secondary or  below | 115 (62.16) | 96 (51.34) | 117 (62.23) |  | 108 (58.06) | 115 (61.83) | 105 (55.85) |  |
| Senior high/ technical  secondary school | 42 (22.70) | 41 (21.93) | 27 (14.36) |  | 44 (23.66) | 32 (17.20) | 34 (18.09) |  |
| Junior college/  university or above | 28 (15.14) | 50 (26.74) | 44 (23.41) |  | 34 (18.28) | 39 (20.97) | 49 (26.06) |  |
| **Income per month (yuan)** |  |  |  | 0.08 |  |  |  | 0.20 |
| < 5000 | 119 (64.33) | 95 (50.80) | 98 (52.13) |  | 109 (58.61) | 107 (57.52) | 96 (51.07) |  |
| 5000 to < 10000 | 44 (23.78) | 60 (32.09) | 59 (31.38) |  | 53 (28.49) | 56 (30.11) | 54 (28.72) |  |
| > 10000 | 22 (11.89) | 32 (17.11) | 31 (16.49) |  | 24 (12.90) | 23 (12.37) | 38 (20.21) |  |

BMI, body mass index; FIGO, The International Federation of Gynecology and Obstetrics; MET, metabolic equivalents of task; TAC, total antioxidant capacity.

Values are numbers (percentages) for categorical variables and median (interquartile range) for continuous variables.

**Supplementary Table S2. General characteristics of 560 patients with ovarian cancer grouped by tertiles of pre-diagnosis and post-diagnosis composite dietary antioxidant index**

| **Characteristic** | **Pre-diagnosis CDAI** | | | ***P* value** | **Post-diagnosis CDAI** | | | ***P* value** |
| --- | --- | --- | --- | --- | --- | --- | --- | --- |
|  | **Tertile 1** | **Tertile 2** | **Tertile 3** |  | **Tertile 1** | **Tertile 2** | **Tertile 3** |  |
| **N (death/total)** | 74/186 | 79/186 | 58/188 | 0.05 | 90/186 | 68/186 | 53/188 | 0.01 |
| **Survival time (months)** | 43.53 (24.03-57.97) | 42.48 (25.73-57.50) | 48.18 (30.42-65.32) | 0.02 | 36.62 (20.40-56.17) | 43.53 (29.17-60.10) | 50.52 (31.82-64.92) | 0.01 |
| **Age at diagnosis (years)** | 53.00 (47.00-58.00) | 53.00 (46.00-59.00) | 54.50 (48.00-61.50) | 0.15 | 54.00 (48.00-60.00) | 52.00 (47.00-59.00) | 54.00 (48.00-60.00) | 0.33 |
| **Total energy intake (kcal/d)** | 970.63 (837.72-1098.68) | 1327.52 (1178.09-1611.35) | 1934.21 (1674.33-2279.63) | 0.01 | 913.10 (730.84-1011.90) | 1230.90 (1086.59-1434.69) | 1715.43 (1545.46-1948.35) | 0.01 |
| **Physical activity (MET*h/d)** | 11.70 (6.10-18.87) | 10.46 (5.41-17.66) | 9.85 (5.60-18.67) | 0.50 | 5.90 (3.60-9.00) | 6.30 (3.60-9.00) | 7.20 (3.60-9.00) | 0.30 |
| **BMI (kg/m^2^)** |  |  |  | 0.80 |  |  |  | 0.05 |
| < 24 | 117 (62.90) | 123 (66.13) | 120 (63.83) |  | 127 (68.28) | 106 (56.99) | 109 (57.98) |  |
| ≥ 24 | 69 (37.10) | 63 (33.87) | 68 (36.17) |  | 59 (31.72) | 80 (43.01) | 79 (42.02) |  |
| **Smoking status** |  |  |  | 0.18 |  |  |  | 0.57 |
| No | 161 (86.56) | 172 (92.47) | 168 (89.36) |  | 165 (88.71) | 163 (87.63) | 171 (90.96) |  |
| Yes | 25 (13.44) | 14 (7.53) | 20 (10.64) |  | 21 (11.29) | 23 (12.37) | 17 (9.04) |  |
| **Alcohol drinking** |  |  |  | 0.01 |  |  |  | 0.42 |
| No | 140 (75.27) | 126 (67.74) | 106 (56.38) |  | 154 (82.80) | 146 (78.49) | 146 (77.66) |  |
| Yes | 46 (24.73) | 60 (32.26) | 82 (43.62) |  | 32 (17.20) | 40 (21.51) | 42 (22.34) |  |
| **Dietary Supplement** |  |  |  | 0.02 |  |  |  | 0.07 |
| No | 158 (84.95) | 152 (81.72) | 139 (73.94) |  | 129 (69.35) | 108 (58.06) | 115 (61.17) |  |
| Yes | 28 (15.05) | 34 (18.28) | 49 (26.06) |  | 57 (30.65) | 78 (41.94) | 73 (38.83) |  |
| **Menopause status** |  |  |  | 0.04 |  |  |  | 0.79 |
| No | 61 (32.80) | 65 (34.95) | 44 (23.40) |  | 55 (29.57) | 60 (32.26) | 55 (29.26) |  |
| Yes | 125 (67.20) | 121 (65.05) | 144 (76.60) |  | 131 (70.43) | 126 (67.74) | 133 (70.74) |  |
| **Parity** |  |  |  | 0.73 |  |  |  | 0.09 |
| ≤ 1 | 130 (69.89) | 135 (72.58) | 138 (73.40) |  | 123 (66.13) | 138 (74.19) | 142 (75.53) |  |
| ≥ 2 | 56 (30.11) | 51 (27.42) | 50 (26.60) |  | 63 (33.87) | 48 (25.81) | 46 (24.47) |  |
| **Histological type** |  |  |  | 0.36 |  |  |  | 0.84 |
| Serous | 133 (71.51) | 145 (77.96) | 141 (75.00) |  | 138 (74.19) | 142 (76.34) | 139 (73.94) |  |
| Non-serous | 53 (28.49) | 41 (22.04) | 47 (25.00) |  | 48 (25.81) | 44 (23.66) | 49 (26.06) |  |
| **Residual lesions** |  |  |  | 0.64 |  |  |  | 0.81 |
| No | 148 (79.57) | 144 (77.42) | 153 (81.38) |  | 145 (77.96) | 150 (80.65) | 150 (79.79) |  |
| Yes | 38 (20.43) | 42 (22.58) | 35 (18.62) |  | 41 (22.04) | 36 (19.35) | 38 (20.21) |  |
| **FIGO stage** |  |  |  | 0.15 |  |  |  | 0.91 |
| I-II | 84 (45.16) | 66 (35.48) | 79 (42.02) |  | 78 (41.94) | 74 (39.78) | 77 (40.96) |  |
| III-IV | 102 (54.84) | 120 (64.52) | 109 (57.98) |  | 108 (58.06) | 112 (60.22) | 111 (59.04) |  |
| **Educational level** |  |  |  | 0.40 |  |  |  | 0.18 |
| Junior secondary or  below | 118 (63.45) | 100 (53.76) | 110 (58.51) |  | 115 (61.82) | 108 (58.06) | 105 (55.85) |  |
| Senior high/ technical  secondary school | 32 (17.20) | 43 (23.12) | 35 (18.62) |  | 40 (21.51) | 38 (20.43) | 32 (17.02) |  |
| Junior college/  university or above | 36 (19.35) | 43 (23.12) | 43 (22.87) |  | 31 (16.67) | 40 (21.51) | 51 (27.13) |  |
| **Income per month (yuan)** |  |  |  | 0.21 |  |  |  | 0.33 |
| < 5000 | 120 (64.51) | 103 (55.38) | 89 (47.34) |  | 109 (58.60) | 103 (55.38) | 100 (53.19) |  |
| 5000 to < 10000 | 43 (23.12) | 53 (28.49) | 67 (35.64) |  | 57 (30.65) | 50 (26.88) | 56 (29.79) |  |
| > 10000 | 23 (12.37) | 30 (16.13) | 32 (17.02) |  | 20 (10.75) | 33 (17.74) | 32 (17.02) |  |

BMI, body mass index; CDAI, composite dietary antioxidant index; FIGO, The International Federation of Gynecology and Obstetrics; MET, metabolic equivalents of task.

Values are numbers (percentages) for categorical variables and median (interquartile range) for continuous variables.

**Supplementary Table S3. General characteristics of 560 patients with ovarian cancer grouped by tertiles of pre-diagnosis and post-diagnosis dietary antioxidant index of Vitamin C**

| **Characteristic** | **Pre-diagnosis Vitamin C** | | | ***P* value** | **Post-diagnosis Vitamin C** | | | ***P* value** |
| --- | --- | --- | --- | --- | --- | --- | --- | --- |
|  | **Tertile 1** | **Tertile 2** | **Tertile 3** |  | **Tertile 1** | **Tertile 2** | **Tertile 3** |  |
| **N (death/total)** | 82/186 | 74/186 | 55/188 | 0.01 | 87/186 | 67/186 | 57/188 | 0.01 |
| **Survival time (months)** | 41.05 (24.17-56.17) | 42.28 (21.80-62.90) | 48.62 (32.85-68.07) | 0.01 | 39.92 (20.37-57.80) | 43.25 (28.93-61.57) | 48.35 (30.92-64.00) | 0.01 |
| **Age at diagnosis (years)** | 53.00 (47.00-59.00) | 53.50 (48.00-59.00) | 52.50 (47.00-60.00) | 0.91 | 53.00 (48.00-59.00) | 52.00 (47.00-59.00) | 54.00 (48.00-60.50) | 0.22 |
| **Total energy intake (kcal/d)** | 1014.05 (858.09-1235.74) | 1379.90 (1146.11-1683.31) | 1809.26 (1392.13-2212.55) | 0.01 | 942.73 (742.94-1094.43) | 1236.27 (1042.32-1531.14) | 1635.69 (1385.36-1905.47) | 0.01 |
| **Physical activity (MET*h/d)** | 11.21 (5.60-17.66) | 10.56 (5.60-17.69) | 10.27 (5.61-18.87) | 0.85 | 6.30 (3.60-9.00) | 6.30 (3.60-9.00) | 7.20 (3.60-9.00) | 0.77 |
| **BMI (kg/m^2^)** |  |  |  | 0.70 |  |  |  | 0.10 |
| < 24 | 119 (63.98) | 116 (62.37) | 125 (66.49) |  | 124 (66.67) | 104 (55.91) | 114 (60.64) |  |
| ≥ 24 | 67 (36.02) | 70 (37.63) | 63 (33.51) |  | 62 (33.33) | 82 (44.09) | 74 (39.36) |  |
| **Smoking status** |  |  |  | 0.43 |  |  |  | 0.98 |
| No | 162 (87.10) | 169 (90.86) | 170 (90.43) |  | 166 (89.25) | 165 (88.71) | 168 (89.36) |  |
| Yes | 24 (12.90) | 17 (9.14) | 18 (9.57) |  | 20 (10.75) | 21 (11.29) | 20 (10.64) |  |
| **Alcohol drinking** |  |  |  | 0.01 |  |  |  | 0.44 |
| No | 144 (77.42) | 118 (63.44) | 110 (58.51) |  | 153 (82.26) | 143 (76.88) | 150 (79.79) |  |
| Yes | 42 (22.58) | 68 (36.56) | 78 (41.49) |  | 33 (17.74) | 43 (23.12) | 38 (20.21) |  |
| **Dietary Supplement** |  |  |  | 0.38 |  |  |  | 0.03 |
| No | 154 (82.80) | 150 (80.65) | 145 (77.13) |  | 131 (70.43) | 109 (58.60) | 112 (59.57) |  |
| Yes | 32 (17.20) | 36 (19.35) | 43 (22.87) |  | 55 (29.57) | 77 (41.40) | 76 (40.43) |  |
| **Menopause status** |  |  |  |  |  |  |  |  |
| No | 126 (67.74) | 123 (66.13) | 141 (75.00) | 0.14 | 130 (69.89) | 122 (65.59) | 138 (73.40) | 0.26 |
| Yes | 60 (32.26) | 63 (33.87) | 47 (25.00) |  | 56 (30.11) | 64 (34.41) | 50 (26.60) |  |
| **Parity** |  |  |  | 0.85 |  |  |  | 0.34 |
| ≤ 1 | 131 (70.43) | 135 (72.58) | 137 (72.87) |  | 127 (68.28) | 135 (72.58) | 141 (75.00) |  |
| ≥ 2 | 55 (29.57) | 51 (27.42) | 51 (27.13) |  | 59 (31.72) | 51 (27.42) | 47 (25.00) |  |
| **Histological type** |  |  |  | 0.08 |  |  |  | 0.81 |
| Serous | 130 (69.89) | 149 (80.11) | 140 (74.47) |  | 141 (75.81) | 136 (73.12) | 142 (75.53) |  |
| Non-serous | 56 (30.11) | 37 (19.89) | 48 (25.53) |  | 45 (24.19) | 50 (26.88) | 46 (24.47) |  |
| **Residual lesions** |  |  |  | 0.98 |  |  |  | 0.27 |
| No | 147 (79.03) | 148 (79.57) | 150 (79.79) |  | 148 (79.57) | 154 (82.80) | 143 (76.06) |  |
| Yes | 39 (20.97) | 38 (20.43) | 38 (20.21) |  | 38 (20.43) | 32 (17.20) | 45 (23.94) |  |
| **FIGO stage** |  |  |  | 0.05 |  |  |  | 0.35 |
| I-II | 85 (45.70) | 63 (33.87) | 81 (43.09) |  | 76 (40.86) | 83 (44.62) | 70 (37.23) |  |
| III-IV | 101 (54.30) | 123 (66.13) | 107 (56.91) |  | 110 (59.14) | 103 (55.38) | 118 (62.77) |  |
| **Educational level** |  |  |  | 0.29 |  |  |  | 0.54 |
| Junior secondary or  below | 118 (63.44) | 101 (54.30) | 109 (57.98) |  | 112 (60.22) | 110 (59.14) | 106 (56.38) |  |
| Senior high/ technical  secondary school | 37 (19.89) | 38 (20.43) | 35 (18.62) |  | 40 (21.51) | 36 (19.35) | 34 (18.09) |  |
| Junior college/  university or above | 31 (16.67) | 47 (25.27) | 44 (23.40) |  | 34 (18.28) | 40 (21.51) | 48 (25.53) |  |
| **Income per month (yuan)** |  |  |  | 0.04 |  |  |  | 0.06 |
| < 5000 | 120 (64.52) | 95 (51.08) | 97 (51.60) |  | 106 (56.99) | 112 (60.22) | 94 (50.00) |  |
| 5000 to < 10000 | 47 (25.27) | 57 (30.65) | 59 (31.38) |  | 56 (30.11) | 53 (28.49) | 54 (28.72) |  |
| > 10000 | 19 (10.22) | 34 (18.28) | 32 (17.02) |  | 24 (12.90) | 21 (11.29) | 40 (21.28) |  |

BMI, body mass index; FIGO, The International Federation of Gynecology and Obstetrics; MET, metabolic equivalents of task.

Values are numbers (percentages) for categorical variables and median (interquartile range) for continuous variables.

**Supplementary Table S4. General characteristics of 560 patients with ovarian cancer grouped by tertiles of pre-diagnosis and post-diagnosis dietary antioxidant index of** **retinol**

| **Characteristic** | **Pre-diagnosis retinol** | | | ***P* value** | **Post-diagnosis retinol** | | | ***P* value** |
| --- | --- | --- | --- | --- | --- | --- | --- | --- |
|  | **Tertile 1** | **Tertile 2** | **Tertile 3** |  | **Tertile 1** | **Tertile 2** | **Tertile 3** |  |
| **N (death/total)** | 76/186 | 70/186 | 65/188 | 0.46 | 85/186 | 62/186 | 64/188 | 0.02 |
| **Survival time (months)** | 43.13 (25.73-58.03) | 42.72 (28.93-60.80) | 47.50 (25.50-63.82) | 0.57 | 36.28 (20.80-55.63) | 45.90 (28.93-58.30) | 48.35 (31.10-67.25) | 0.01 |
| **Age at diagnosis (years)** | 52.00 (48.00-59.00) | 54.00 (47.00-61.00) | 53.00 (47.00-59.00) | 0.72 | 53.00 (48.00-59.00) | 54.00 (48.00-60.00) | 52.50 (46.50-59.00) | 0.22 |
| **Total energy intake (kcal/d)** | 1069.54 (864.71-1234.66) | 1523.67 (1284.96-1804.93) | 1641.18 (1132.33-2159.87) | 0.01 | 1019.15 (778.07-1281.54) | 1381.17 (1026.28-1693.06) | 1414.93 (1116.40-1770.00) | 0.01 |
| **Physical activity (MET*h/d)** | 11.70 (5.60-18.04) | 9.81 (5.60-18.85) | 11.20 (5.95-17.09) | 0.98 | 5.70 (3.60-9.00) | 7.20 (3.60-9.86) | 7.20 (3.60-9.00) | 0.15 |
| **BMI (kg/m^2^)** |  |  |  | 0.48 |  |  |  | 0.31 |
| < 24 | 117 (62.90) | 126 (67.74) | 117 (62.23) |  | 120 (64.52) | 115 (61.83) | 107 (56.91) |  |
| ≥ 24 | 69 (37.10) | 60 (32.26) | 71 (37.77) |  | 66 (35.48) | 71 (38.17) | 81 (43.09) |  |
| **Smoking status** |  |  |  | 0.04 |  |  |  | 0.03 |
| No | 162 (87.10) | 175 (94.09) | 164 (87.23) |  | 160 (86.02) | 175 (94.09) | 164 (87.23) |  |
| Yes | 24 (12.90) | 11 (5.91) | 24 (12.77) |  | 26 (13.98) | 11 (5.91) | 24 (12.77) |  |
| **Alcohol drinking** |  |  |  | 0.01 |  |  |  | 0.05 |
| No | 136 (73.12) | 130 (69.89) | 106 (56.38) |  | 155 (83.33) | 152 (81.72) | 139 (73.94) |  |
| Yes | 50 (26.88) | 56 (30.11) | 82 (43.62) |  | 31 (16.67) | 34 (18.28) | 49 (26.06) |  |
| **Dietary Supplement** |  |  |  | 0.19 |  |  |  | 0.01 |
| No | 157 (84.41) | 147 (79.03) | 145 (77.13) |  | 137 (73.66) | 117 (62.90) | 98 (52.13) |  |
| Yes | 29 (15.59) | 39 (20.97) | 43 (22.87) |  | 49 (26.34) | 69 (37.10) | 90 (47.87) |  |
| **Menopause status** |  |  |  |  |  |  |  |  |
| No | 127 (68.28) | 135 (72.58) | 128 (68.09) | 0.57 | 130 (69.89) | 128 (68.82) | 132 (70.21) | 0.95 |
| Yes | 59 (31.72) | 51 (27.42) | 60 (31.91) |  | 56 (30.11) | 58 (31.18) | 56 (29.79) |  |
| **Parity** |  |  |  | 0.81 |  |  |  | 0.41 |
| ≤ 1 | 134 (72.04) | 131 (70.43) | 138 (73.40) |  | 130 (69.89) | 131 (70.43) | 142 (75.53) |  |
| ≥ 2 | 52 (27.96) | 55 (29.57) | 50 (26.60) |  | 56 (30.11) | 55 (29.57) | 46 (24.47) |  |
| **Histological type** |  |  |  | 0.81 |  |  |  | 0.93 |
| Serous | 139 (74.73) | 142 (76.34) | 138 (73.40) |  | 141 (75.81) | 138 (74.19) | 140 (74.47) |  |
| Non-serous | 47 (25.27) | 44 (23.66) | 50 (26.60) |  | 45 (24.19) | 48 (25.81) | 48 (25.53) |  |
| **Residual lesions** |  |  |  | 0.59 |  |  |  | 0.95 |
| No | 146 (78.49) | 145 (77.96) | 154 (81.91) |  | 148 (79.57) | 149 (80.11) | 148 (78.72) |  |
| Yes | 40 (21.51) | 41 (22.04) | 34 (18.09) |  | 38 (20.43) | 37 (19.89) | 40 (21.28) |  |
| **FIGO stage** |  |  |  | 0.91 |  |  |  | 0.62 |
| I-II | 76 (40.86) | 74 (39.78) | 79 (42.02) |  | 71 (38.17) | 80 (43.01) | 78 (41.49) |  |
| III-IV | 110 (59.14) | 112 (60.22) | 109 (57.98) |  | 115 (61.83) | 106 (56.99) | 110 (58.51) |  |
| **Educational level** |  |  |  | 0.03 |  |  |  | 0.69 |
| Junior secondary or  below | 120 (64.52) | 105 (56.45) | 103 (54.79) |  | 110 (59.14) | 106 (56.99) | 112 (59.57) |  |
| Senior high/ technical  secondary school | 35 (18.82) | 44 (23.66) | 31 (16.49) |  | 41 (22.04) | 36 (19.35) | 33 (17.55) |  |
| Junior college/  university or above | 31 (16.67) | 37 (19.89) | 54 (28.72) |  | 35 (18.82) | 44 (23.66) | 43 (22.87) |  |
| **Income per month (yuan)** |  |  |  | 0.09 |  |  |  | 0.68 |
| < 5000 | 115 (61.83) | 102 (54.84) | 95 (50.53) |  | 102 (54.84) | 108 (58.06) | 102 (54.26) |  |
| 5000 to < 10000 | 51 (27.42) | 57 (30.65) | 55 (29.26) |  | 58 (31.18) | 53 (28.49) | 52 (27.66) |  |
| > 10000 | 20 (10.75) | 27 (14.52) | 38 (20.21) |  | 26 (13.98) | 25 (13.44) | 34 (18.09) |  |

BMI, body mass index; FIGO, The International Federation of Gynecology and Obstetrics; MET, metabolic equivalents of task.

Values are numbers (percentages) for categorical variables and median (interquartile range) for continuous variables.

**Supplementary Table S5. General characteristics of 560 patients with ovarian cancer grouped by tertiles of pre-diagnosis and post-diagnosis dietary antioxidant index of α-carotene**

| **Characteristic** | **Pre-diagnosis α-carotene** | | | ***P* value** | **Post-diagnosis α-carotene** | | | ***P* value** |
| --- | --- | --- | --- | --- | --- | --- | --- | --- |
|  | **Tertile 1** | **Tertile 2** | **Tertile 3** |  | **Tertile 1** | **Tertile 2** | **Tertile 3** |  |
| **N (death/total)** | 73/186 | 68/186 | 70/188 | 0.86 | 82/186 | 69/186 | 60/188 | 0.05 |
| **Survival time (months)** | 44.12 (25.33-61.80) | 44.43 (28.83-58.30) | 44.68 (27.07-64.00) | 0.84 | 41.88 (22.13-64.57) | 42.60 (27.20-55.43) | 45.70 (31.65-63.15) | 0.14 |
| **Age at diagnosis (years)** | 53.00 (47.00-58.00) | 53.00 (47.00-59.00) | 54.00 (48.00-61.50) | 0.30 | 53.00 (47.00-59.00) | 54.00 (48.00-60.00) | 53.00 (47.00-60.00) | 0.74 |
| **Total energy intake (kcal/d)** | 1128.01 (879.92-1450.48) | 1345.90 (1051.41-1800.73) | 1624.85 (1277.72-2021.53) | 0.01 | 1040.05 (794.54-1317.71) | 1209.38 (965.85-1589.36) | 1520.85 (1181.79-1796.95) | 0.01 |
| **Physical activity (MET*h/d)** | 10.40 (5.18-17.46) | 9.95 (5.84-18.04) | 11.46 (5.70-18.99) | 0.49 | 7.16 (3.60-9.40) | 5.79 (3.60-9.00) | 7.20 (3.60-9.00) | 0.38 |
| **BMI (kg/m^2^)** |  |  |  | 0.01 |  |  |  | 0.62 |
| < 24 | 100 (53.76) | 129 (69.35) | 131 (69.68) |  | 114 (61.29) | 118 (63.44) | 110 (58.51) |  |
| ≥ 24 | 86 (46.24) | 57 (30.65) | 57 (30.32) |  | 72 (38.71) | 68 (36.56) | 78 (41.49) |  |
| **Smoking status** |  |  |  | 0.72 |  |  |  | 0.57 |
| No | 166 (89.25) | 169 (90.86) | 166 (88.30) |  | 165 (88.71) | 163 (87.63) | 171 (90.96) |  |
| Yes | 20 (10.75) | 17 (9.14) | 22 (11.70) |  | 21 (11.29) | 23 (12.37) | 17 (9.04) |  |
| **Alcohol drinking** |  |  |  | 0.30 |  |  |  | 0.18 |
| No | 120 (64.52) | 119 (63.98) | 133 (70.74) |  | 148 (79.57) | 141 (75.81) | 157 (83.51) |  |
| Yes | 66 (35.48) | 67 (36.02) | 55 (29.26) |  | 38 (20.43) | 45 (24.19) | 31 (16.49) |  |
| **Dietary Supplement** |  |  |  | 0.26 |  |  |  | 0.20 |
| No | 155 (83.33) | 150 (80.65) | 144 (76.60) |  | 124 (66.67) | 119 (63.98) | 109 (57.98) |  |
| Yes | 31 (16.67) | 36 (19.35) | 44 (23.40) |  | 62 (33.33) | 67 (36.02) | 79 (42.02) |  |
| **Menopause status** |  |  |  |  |  |  |  |  |
| No | 123 (66.13) | 128 (68.82) | 139 (73.94) | 0.25 | 125 (67.20) | 130 (69.89) | 135 (71.81) | 0.62 |
| Yes | 63 (33.87) | 58 (31.18) | 49 (26.06) |  | 61 (32.80) | 56 (30.11) | 53 (28.19) |  |
| **Parity** |  |  |  | 0.50 |  |  |  | 0.60 |
| ≤ 1 | 137 (73.66) | 128 (68.82) | 138 (73.40) |  | 135 (72.58) | 129 (69.35) | 139 (73.94) |  |
| ≥ 2 | 49 (26.34) | 58 (31.18) | 50 (26.60) |  | 51 (27.42) | 57 (30.65) | 49 (26.06) |  |
| **Histological type** |  |  |  | 0.02 |  |  |  | 0.33 |
| Serous | 126 (67.74) | 144 (77.42) | 149 (79.26) |  | 133 (71.51) | 139 (74.73) | 147 (78.19) |  |
| Non-serous | 60 (32.26) | 42 (22.58) | 39 (20.74) |  | 53 (28.49) | 47 (25.27) | 41 (21.81) |  |
| **Residual lesions** |  |  |  | 1.00 |  |  |  | 0.70 |
| No | 148 (79.57) | 148 (79.57) | 149 (79.26) |  | 148 (79.57) | 151 (81.18) | 146 (77.66) |  |
| Yes | 38 (20.43) | 38 (20.43) | 39 (20.74) |  | 38 (20.43) | 35 (18.82) | 42 (22.34) |  |
| **FIGO stage** |  |  |  | 0.54 |  |  |  | 0.35 |
| I-II | 82 (44.09) | 72 (38.71) | 75 (39.89) |  | 79 (42.47) | 81 (43.55) | 69 (36.70) |  |
| III-IV | 104 (55.91) | 114 (61.29) | 113 (60.11) |  | 107 (57.53) | 105 (56.45) | 119 (63.30) |  |
| **Educational level** |  |  |  | 0.48 |  |  |  | 0.98 |
| Junior secondary or  below | 109 (58.60) | 101 (54.30) | 118 (62.77) |  | 107 (57.53) | 111 (59.68) | 110 (58.51) |  |
| Senior high/ technical  secondary school | 34 (18.28) | 43 (23.12) | 33 (17.55) |  | 38 (20.43) | 34 (18.28) | 38 (20.21) |  |
| Junior college/  university or above | 43 (23.12) | 42 (22.58) | 37 (19.68) |  | 41 (22.04) | 41 (22.04) | 40 (21.28) |  |
| **Income per month (yuan)** |  |  |  | 0.02 |  |  |  | 0.95 |
| < 5000 | 104 (55.91) | 119 (63.98) | 89 (47.34) |  | 108 (58.06) | 102 (54.84) | 102 (54.26) |  |
| 5000 to < 10000 | 54 (29.03) | 41 (22.04) | 68 (36.17) |  | 52 (27.96) | 55 (29.57) | 56 (29.79) |  |
| > 10000 | 28 (15.05) | 26 (13.98) | 31 (16.49) |  | 26 (13.98) | 29 (15.59) | 30 (15.96) |  |

BMI, body mass index; FIGO, The International Federation of Gynecology and Obstetrics; MET, metabolic equivalents of task.

Values are numbers (percentages) for categorical variables and median (interquartile range) for continuous variables.

**Supplementary Table S6. General characteristics of 560 patients with ovarian cancer grouped by tertiles of pre-diagnosis and post-diagnosis dietary antioxidant index of β-carotene**

| **Characteristic** | **Pre-diagnosis β-carotene** | | | ***P* value** | **Post-diagnosis β-carotene** | | | ***P* value** |
| --- | --- | --- | --- | --- | --- | --- | --- | --- |
|  | **Tertile 1** | **Tertile 2** | **Tertile 3** |  | **Tertile 1** | **Tertile 2** | **Tertile 3** |  |
| **N (death/total)** | 67/185 | 80/187 | 64/188 | 0.19 | 87/186 | 67/186 | 57/188 | 0.01 |
| **Survival time (months)** | 41.33 (27.00-59.87) | 43.47 (22.83-57.83) | 48.10 (29.03-68.07) | 0.07 | 37.23 (20.37-57.80) | 45.15 (30.57-63.83) | 46.87 (30.20-63.18) | 0.01 |
| **Age at diagnosis (years)** | 53.00 (46.00-58.00) | 53.00 (48.00-59.00) | 54.00 (48.00-61.00) | 0.12 | 53.00 (48.00-59.00) | 53.00 (48.00-59.00) | 54.00 (47.00-61.00) | 0.80 |
| **Total energy intake (kcal/d)** | 1060.46 (872.91-1371.93) | 1400.78 (1136.08-1692.71) | 1703.22 (1279.26-2096.09) | 0.01 | 979.42 (778.07-1201.39) | 1263.53 (1001.81-1589.36) | 1547.01 (1231.87-1839.24) | 0.01 |
| **Physical activity (MET*h/d)** | 11.69 (6.10-17.80) | 9.80 (5.60-16.87) | 11.68 (5.61-19.93) | 0.24 | 6.50 (3.60-9.00) | 6.71 (3.60-9.40) | 7.16 (3.60-9.00) | 0.93 |
| **BMI (kg/m^2^)** |  |  |  | 0.03 |  |  |  | 0.43 |
| < 24 | 105 (56.76) | 128 (68.45) | 127 (67.55) |  | 120 (64.52) | 113 (60.75) | 109 (57.98) |  |
| ≥ 24 | 80 (43.24) | 59 (31.55) | 61 (32.45) |  | 66 (35.48) | 73 (39.25) | 79 (42.02) |  |
| **Smoking status** |  |  |  | 0.28 |  |  |  | 0.10 |
| No | 167 (90.27) | 162 (86.63) | 172 (91.49) |  | 161 (86.56) | 173 (93.01) | 165 (87.77) |  |
| Yes | 18 (9.73) | 25 (13.37) | 16 (8.51) |  | 25 (13.44) | 13 (6.99) | 23 (12.23) |  |
| **Alcohol drinking** |  |  |  | 0.73 |  |  |  | 0.71 |
| No | 127 (68.65) | 123 (65.78) | 122 (64.89) |  | 148 (79.57) | 145 (77.96) | 153 (81.38) |  |
| Yes | 58 (31.35) | 64 (34.22) | 66 (35.11) |  | 38 (20.43) | 41 (22.04) | 35 (18.62) |  |
| **Dietary Supplement** |  |  |  | 0.15 |  |  |  | 0.14 |
| No | 157 (84.86) | 145 (77.54) | 147 (78.19) |  | 127 (68.28) | 109 (58.60) | 116 (61.70) |  |
| Yes | 28 (15.14) | 42 (22.46) | 41 (21.81) |  | 59 (31.72) | 77 (41.40) | 72 (38.30) |  |
| **Menopause status** |  |  |  |  |  |  |  |  |
| No | 120 (64.86) | 126 (67.38) | 144 (76.60) | 0.03 | 123 (66.13) | 137 (73.66) | 130 (69.15) | 0.28 |
| Yes | 65 (35.14) | 61 (32.62) | 44 (23.40) |  | 63 (33.87) | 49 (26.34) | 58 (30.85) |  |
| **Parity** |  |  |  | 0.52 |  |  |  | 0.29 |
| ≤ 1 | 132 (71.35) | 140 (74.87) | 131 (69.68) |  | 137 (73.66) | 126 (67.74) | 140 (74.47) |  |
| ≥ 2 | 53 (28.65) | 47 (25.13) | 57 (30.32) |  | 49 (26.34) | 60 (32.26) | 48 (25.53) |  |
| **Histological type** |  |  |  | 0.41 |  |  |  | 0.44 |
| Serous | 132 (71.35) | 143 (76.47) | 144 (76.60) |  | 133 (71.51) | 142 (76.34) | 144 (76.60) |  |
| Non-serous | 53 (28.65) | 44 (23.53) | 44 (23.40) |  | 53 (28.49) | 44 (23.66) | 44 (23.40) |  |
| **Residual lesions** |  |  |  | 0.95 |  |  |  | 0.27 |
| No | 148 (80) | 149 (79.68) | 148 (78.72) |  | 149 (80.11) | 141 (75.81) | 155 (82.45) |  |
| Yes | 37 (20) | 38 (20.32) | 40 (21.28) |  | 37 (19.89) | 45 (24.19) | 33 (17.55) |  |
| **FIGO stage** |  |  |  | 0.86 |  |  |  | 0.21 |
| I-II | 78 (42.16) | 77 (41.18) | 74 (39.36) |  | 85 (45.70) | 75 (40.32) | 69 (36.70) |  |
| III-IV | 107 (57.84) | 110 (58.82) | 114 (60.64) |  | 101 (54.30) | 111 (59.68) | 119 (63.30) |  |
| **Educational level** |  |  |  | 0.50 |  |  |  | 0.86 |
| Junior secondary or  below | 107 (57.84) | 109 (58.29) | 112 (59.57) |  | 115 (61.83) | 107 (57.53) | 106 (56.38) |  |
| Senior high/ technical  secondary school | 36 (19.46) | 43 (22.99) | 31 (16.49) |  | 34 (18.28) | 37 (19.89) | 39 (20.74) |  |
| Junior college/  university or above | 42 (22.70) | 35 (18.72) | 45 (23.94) |  | 37 (19.89) | 42 (22.58) | 43 (22.87) |  |
| **Income per month (yuan)** |  |  |  | 0.24 |  |  |  | 0.13 |
| < 5000 | 114 (61.62) | 103 (55.08) | 95 (50.53) |  | 113 (60.75) | 102 (54.84) | 97 (51.60) |  |
| 5000 to < 10000 | 49 (26.49) | 56 (29.95) | 58 (30.85) |  | 50 (26.88) | 60 (32.26) | 53 (28.19) |  |
| > 10000 | 22 (11.89) | 28 (14.97) | 35 (18.62) |  | 23 (12.37) | 24 (12.90) | 38 (20.21) |  |

BMI, body mass index; FIGO, The International Federation of Gynecology and Obstetrics; MET, metabolic equivalents of task.

Values are numbers (percentages) for categorical variables and median (interquartile range) for continuous variables.

**Supplementary Table S7. Associations (hazard ratio and 95% confidence interval) between overall survival of ovarian cancer and changes in cumulative and individual dietary antioxidant nutrients intake from pre-diagnosis to post-diagnosis**

|  | **Pre-Post** | | | | | | | | |
| --- | --- | --- | --- | --- | --- | --- | --- | --- | --- |
|  | **Low–Low** | **Low–Medium** | **Low–High** | **Medium-Low** | **Medium-Medium** | **Medium-High** | **High–Low** | **High–Medium** | **High–High** |
| **TAC** |  |  |  |  |  |  |  |  |  |
| N (death/total) | 47/98 | 16/47 | 21/40 | 28/53 | 25/88 | 13/46 | 15/35 | 20/51 | 26/102 |
| HR (95% CI) | 1.00 (ref.) | 0.71 (0.38, 1.32) | 0.68 (0.37, 1.22) | 1.16 (0.63, 2.11) | 0.53 (0.29, 0.97) | 0.72 (0.34, 1.53) | 0.74 (0.30, 1.82) | 0.77 (0.32, 1.85) | 0.40 (0.16, 0.94) |
| **CDAI** |  |  |  |  |  |  |  |  |  |
| N (death/total) | 42/97 | 18/55 | 14/34 | 34/55 | 30/82 | 15/49 | 14/34 | 20/49 | 24/105 |
| HR (95% CI) | 1.00 (ref.) | 0.83 (0.45, 1.52) | 0.77 (0.36, 1.64) | 0.94 (0.46, 1.93) | 0.61 (0.33, 1.15) | 0.49 (0.21, 1.14) | 0.76 (0.27, 2.13) | 0.84 (0.31, 2.26) | 0.33 (0.12, 0.88) |
| **Vitamin C** |  |  |  |  |  |  |  |  |  |
| N (death/total) | 44/100 | 21/50 | 17/36 | 27/51 | 30/84 | 17/51 | 16/35 | 16/52 | 23/101 |
| HR (95% CI) | 1.00 (ref.) | 2.89 (1.12, 7.50) | 1.63 (0.52, 5.12) | 0.64 (0.30, 1.37) | 1.57 (0.64, 3.85) | 0.92 (0.32, 2.67) | 0.58 (0.21, 1.59) | 0.46 (0.16, 1.32) | 0.51 (0.18, 1.42) |
| **Retinol** |  |  |  |  |  |  |  |  |  |
| N (death/total) | 35/80 | 26/64 | 15/42 | 23/52 | 25/63 | 22/71 | 29/54 | 16/59 | 20/75 |
| HR (95% CI) | 1.00 (ref.) | 2.35 (0.51, 10.89) | 1.48 (0.40, 5.43) | 0.77 (0.36, 1.63) | 1.32 (0.28, 6.17) | 1.04 (0.29, 3.77) | 1.57 (0.64, 3.83) | 0.74 (0.15, 3.64) | 0.87 (0.21, 3.73) |
| **α-carotene** |  |  |  |  |  |  |  |  |  |
| N (death/total) | 38/88 | 21/53 | 14/45 | 23/48 | 24/76 | 21/62 | 26/50 | 22/57 | 22/81 |
| HR (95% CI) | 1.00 (ref.) | 2.02 (0.43, 9.56) | 1.19 (0.34, 4.11) | 1.16 (0.57, 2.37) | 1.33 (0.32, 5.51) | 2.22 (0.58, 8.45) | 1.81 (0.77, 4.23) | 1.58 (0.37, 6.77) | 1.62 (0.44, 5.96) |
| **β-carotene** |  |  |  |  |  |  |  |  |  |
| N (death/total) | 40/89 | 17/59 | 10/37 | 32/61 | 25/62 | 23/64 | 15/36 | 25/65 | 24/87 |
| HR (95% CI) | 1.00 (ref.) | 0.75 (0.15, 3.82) | 1.55 (0.40, 6.04) | 0.67 (0.32, 1.41) | 1.17 (0.24, 5.73) | 1.27 (0.40, 4.03) | 0.94 (0.33, 2.66) | 1.61 (0.36, 7.18) | 1.70 (0.44, 6.62) |

CDAI, composite dietary antioxidant index; Pre-Post, pre- to post-diagnosis; Ref, reference; TAC, total antioxidant capacity.

A cross-classified change model was utilized to categorize total antioxidant capacity into nine groups based on changes from pre- to post-diagnosis: three consistent groups [consistently low (reference), consistently medium, consistently high] and six inconsistent groups (low to medium; low to high; medium to low; medium to high; high to low; high to medium).

* TAC adjusted for age at diagnosis, pre-diagnosis and change in body mass index, pre-diagnosis and change in total energy intake, pre-diagnosis and change in physical activity, income, education, pre-diagnosis and change in smoking status, pre-diagnosis and change in alcohol drinking, pre-diagnosis and change in tea drinking, pre-diagnosis and change in dietary supplement, menopause status, parity, histological type, residual lesions, the International Federation of Gynecology and Obstetrics stage, and pre-diagnosis total antioxidant capacity.

CDAI adjusted for age at diagnosis, pre-diagnosis and change in body mass index, pre-diagnosis and change in total energy intake, pre-diagnosis and change in physical activity, income, education, pre-diagnosis and change in smoking status, pre-diagnosis and change in alcohol drinking, pre-diagnosis and change in dietary supplement, menopause status, parity, histological type, residual lesions, the International Federation of Gynecology and Obstetrics stage, and pre-diagnosis composite dietary antioxidant index.

Individual dietary antioxidant nutrients intake adjusted for age at diagnosis, pre-diagnosis and change in body mass index, pre-diagnosis and change in total energy intake, pre-diagnosis and change in physical activity, income, education, pre-diagnosis and change in smoking status, pre-diagnosis and change in alcohol drinking, pre-diagnosis and change in dietary supplement, menopause status, parity, histological type, residual lesions, the International Federation of Gynecology and Obstetrics stage, pre-diagnosis dietary antioxidant index of the nutrient, and pre-diagnosis and change in dietary antioxidant index of the other nutrient.

**Supplementary Table S8. Subgroup analyses for patients with ovarian cancer according to pre-diagnosis and post-diagnosis total antioxidant capacity and composite dietary antioxidant index**

|  | **Deaths/** | **Pre-diagnosis** 1 | | | ***P***mul3 | ***P***add 4 | **Deaths/** | **Post-diagnosis** 2 | | | ***P*** mul3 | ***P***add 4 |
| --- | --- | --- | --- | --- | --- | --- | --- | --- | --- | --- | --- | --- |
|  | **Total** | **T**ertile 1 | **T**ertile 2 | **T**ertile 3 |  |  | **Total** | **T**ertile 1 | **T**ertile 2 | **T**ertile 3 |  |  |
| **TAC** | | | | | | | | | | | | |
| Age at diagnosis |  |  |  |  | 0.51 | 0.96 |  |  |  |  | 0.39 | 0.94 |
| ≤ 50 | 77/209 | 1.00 (ref.) | 0.97 (0.53, 1.79) | 0.49 (0.24, 1.03) |  |  | 77/209 | 1.00 (ref.) | 0.63 (0.35, 1.14) | 0.35 (0.16, 0.74) |  |  |
| > 50 | 134/351 | 1.00 (ref.) | 0.94 (0.60, 1.47) | 0.70 (0.41, 1.21) |  |  | 134/351 | 1.00 (ref.) | 0.66 (0.42, 1.03) | 0.72 (0.42, 1.24) |  |  |
| FIGO stage |  |  |  |  | 0.52 | 0.99 |  |  |  |  | 0.48 | 0.49 |
| I-II | 48/229 | 1.00 (ref.) | 0.33 (0.15, 0.76) | 0.28 (0.10, 0.76) |  |  | 48/229 | 1.00 (ref.) | 0.62 (0.28, 1.36) | 0.68 (0.25, 1.85) |  |  |
| III-IV | 163/331 | 1.00 (ref.) | 1.14 (0.77, 1.70) | 0.72 (0.44, 1.18) |  |  | 163/331 | 1.00 (ref.) | 0.62 (0.41, 0.93) | 0.48 (0.29, 0.79) |  |  |
| Histological type |  |  |  |  | 0.15 | 0.30 |  |  |  |  | 0.15 | 0.92 |
| Serous | 171/419 | 1.00 (ref.) | 0.99 (0.67, 1.45) | 0.66 (0.41, 1.06) |  |  | 171/419 | 1.00 (ref.) | 0.66 (0.45, 0.97) | 0.57 (0.36, 0.92) |  |  |
| Non-serous | 40/141 | 1.00 (ref.) | 0.42 (0.18, 0.97) | 0.30 (0.10, 0.87) |  |  | 40/141 | 1.00 (ref.) | 0.73 (0.27, 1.93) | 0.56 (0.17, 1.83) |  |  |
| Smoking status |  |  |  |  | 0.07 | 0.03 |  |  |  |  | 0.11 | 0.81 |
| No | 188/501 | 1.00 (ref.) | 0.94 (0.65, 1.35) | 0.64 (0.41, 0.99) |  |  | 186/499 | 1.00 (ref.) | 0.68 (0.47, 0.98) | 0.60 (0.38, 0.95) |  |  |
| Yes | 23/59 | 1.00 (ref.) | 0.86 (0.16, 4.68) | 0.26 (0.03, 1.93) |  |  | 25/61 | 1.00 (ref.) | 0.37 (0.10, 1.44) | 0.16 (0.02, 1.23) |  |  |
| Alcohol drinking |  |  |  |  | 0.75 | 0.35 |  |  |  |  | 0.29 | 0.02 |
| No | 142/372 | 1.00 (ref.) | 0.83 (0.55, 1.27) | 0.58 (0.34, 0.98) |  |  | 176/446 | 1.00 (ref.) | 0.74 (0.50, 1.09) | 0.54 (0.33, 0.86) |  |  |
| Yes | 69/188 | 1.00 (ref.) | 0.73 (0.37, 1.44) | 0.43 (0.19, 0.98) |  |  | 35/114 | 1.00 (ref.) | 0.26 (0.08, 0.84) | 0.42 (0.11, 1.60) |  |  |
| Tea drinking |  |  |  |  | 0.27 | 0.07 |  |  |  |  | 0.20 | 0.95 |
| No | 120/308 | 1.00 (ref.) | 0.83 (0.53, 1.29) | 0.49 (0.28, 0.87) |  |  | 136/336 | 1.00 (ref.) | 0.70 (0.45, 1.08) | 0.66 (0.37, 1.15) |  |  |
| Yes | 91/252 | 1.00 (ref.) | 0.82 (0.47, 1.43) | 0.70 (0.35, 1.38) |  |  | 75/224 | 1.00 (ref.) | 0.58 (0.31, 1.10) | 0.60 (0.29, 1.23) |  |  |
| Dietary Supplement |  |  |  |  | 0.74 | 0.50 |  |  |  |  | 0.20 | 0.18 |
| No | 174/449 | 1.00 (ref.) | 0.82 (0.57, 1.19) | 0.53 (0.33, 0.84) |  |  | 145/352 | 1.00 (ref.) | 0.60 (0.40, 0.91) | 0.41 (0.23, 0.71) |  |  |
| Yes | 37/111 | 1.00 (ref.) | 1.20 (0.38, 3.75) | 1.07 (0.31, 3.67) |  |  | 66/208 | 1.00 (ref.) | 0.51 (0.24, 1.12) | 0.84 (0.38, 1.87) |  |  |
| **CDAI** | | | | | | | | | | | | |
| Age at diagnosis |  |  |  |  | 0.01 | 0.05 |  |  |  |  | 0.53 | 0.57 |
| ≤ 50 | 77/209 | 1.00 (ref.) | 1.31 (0.70, 2.48) | 1.45 (0.54, 3.87) |  |  | 77/209 | 1.00 (ref.) | 0.72 (0.36, 1.44) | 0.57 (0.21, 1.56) |  |  |
| > 50 | 134/351 | 1.00 (ref.) | 0.88 (0.55, 1.40) | 0.49 (0.24, 1.02) |  |  | 134/351 | 1.00 (ref.) | 0.74 (0.47, 1.16) | 0.54 (0.27, 1.08) |  |  |
| FIGO stage |  |  |  |  | 0.83 | 0.96 |  |  |  |  | 0.99 | 0.60 |
| I-II | 48/229 | 1.00 (ref.) | 0.37 (0.15, 0.89) | 0.44 (0.14, 1.38) |  |  | 48/229 | 1.00 (ref.) | 0.71 (0.31, 1.64) | 0.70 (0.21, 2.30) |  |  |
| III-IV | 163/331 | 1.00 (ref.) | 1.44 (0.94, 2.20) | 0.77 (0.39, 1.52) |  |  | 163/331 | 1.00 (ref.) | 0.75 (0.48, 1.17) | 0.52 (0.27, 1.00) |  |  |
| Histological type |  |  |  |  | 0.24 | 0.33 |  |  |  |  | 0.64 | 0.73 |
| Serous | 171/419 | 1.00 (ref.) | 1.44 (0.95, 2.16) | 0.81 (0.43, 1.52) |  |  | 171/419 | 1.00 (ref.) | 0.74 (0.49, 1.11) | 0.51 (0.27, 0.96) |  |  |
| Non-serous | 40/141 | 1.00 (ref.) | 0.32 (0.12, 0.83) | 0.46 (0.12, 1.78) |  |  | 40/141 | 1.00 (ref.) | 0.92 (0.32, 2.59) | 0.67 (0.16, 2.83) |  |  |
| Smoking status |  |  |  |  | 0.15 | 0.45 |  |  |  |  | 0.47 | 0.94 |
| No | 188/501 | 1.00 (ref.) | 1.11 (0.75, 1.64) | 0.76 (0.42, 1.38) |  |  | 186/499 | 1.00 (ref.) | 0.82 (0.54, 1.22) | 0.70 (0.39, 1.24) |  |  |
| Yes | 23/59 | 1.00 (ref.) | 3.06 (0.59, 15.89) | 1.58 (0.24, 10.61) |  |  | 25/61 | 1.00 (ref.) | 0.37 (0.11, 1.24) | 0.04 (0.01, 0.57) |  |  |
| Alcohol drinking |  |  |  |  | 0.09 | 0.51 |  |  |  |  | 0.97 | 0.01 |
| No | 142/372 | 1.00 (ref.) | 0.89 (0.58, 1.38) | 0.36 (0.18, 0.76) |  |  | 176/446 | 1.00 (ref.) | 0.83 (0.55, 1.25) | 0.53 (0.29, 0.97) |  |  |
| Yes | 69/188 | 1.00 (ref.) | 1.58 (0.74, 3.40) | 1.81 (0.67, 4.89) |  |  | 35/114 | 1.00 (ref.) | 0.27 (0.08, 0.90) | 0.27 (0.04, 1.76) |  |  |
| Dietary Supplement |  |  |  |  | 0.67 | 0.27 |  |  |  |  | 0.75 | 0.48 |
| No | 174/449 | 1.00 (ref.) | 1.08 (0.73, 1.60) | 0.56 (0.30, 1.07) |  |  | 145/352 | 1.00 (ref.) | 0.68 (0.43, 1.08) | 0.48 (0.23, 0.98) |  |  |
| Yes | 37/111 | 1.00 (ref.) | 0.59 (0.20, 1.70) | 0.54 (0.15, 1.94) |  |  | 66/208 | 1.00 (ref.) | 1.08 (0.53, 2.21) | 0.89 (0.33, 2.38) |  |  |

CDAI, composite dietary antioxidant index; FIGO, The International Federation of Gynecology and Obstetrics; Ref, reference; TAC, total antioxidant capacity.

All hazard ratios (95% confidence intervals) were calculated using Cox proportional hazard models.

^1^ Pre-diagnosis model for TAC adjusted for age at diagnosis, pre-diagnosis BMI, pre-diagnosis total energy intake, pre-diagnosis physical activity, income, education, pre-diagnosis smoking status, pre-diagnosis alcohol drinking, pre-diagnosis tea drinking, pre-diagnosis dietary supplement, menopause status, parity, histological type, residual lesions, and FIGO stage. Pre-diagnosis model for CDAI adjusted for age at diagnosis, pre-diagnosis BMI, pre-diagnosis total energy intake, pre-diagnosis physical activity, income, education, pre-diagnosis smoking status, pre-diagnosis alcohol drinking, pre-diagnosis dietary supplement, menopause status, parity, histological type, residual lesions, and FIGO stage.

^2^ Post-diagnosis model for TAC adjusted for age at diagnosis, post-diagnosis BMI, post-diagnosis total energy intake, post-diagnosis physical activity, income, education, post-diagnosis smoking status, post-diagnosis alcohol drinking, post-diagnosis tea drinking, post-diagnosis dietary supplement, menopause status, parity, histological type, residual lesions, and FIGO stage. Post-diagnosis model for CDAI adjusted for age at diagnosis, post-diagnosis BMI, post-diagnosis total energy intake, post-diagnosis physical activity, income, education, post-diagnosis smoking status, post-diagnosis alcohol drinking, post-diagnosis dietary supplement, menopause status, parity, histological type, residual lesions, and FIGO stage.

^3^ Indicated *P* value for multiplicative interaction.

^4^ Indicated *P* value for additive interaction.

**Supplementary Table S9. Subgroup analyses for patients with ovarian cancer according to pre-diagnosis and post-diagnosis** **individual dietary antioxidant nutrients intake**

|  | **Deaths/** | **Pre-diagnosis** 1 | | | ***P***mul3 | ***P***add 4 | **Deaths/** | **Post-diagnosis** 2 | | | ***P*** mul3 | ***P***add 4 |
| --- | --- | --- | --- | --- | --- | --- | --- | --- | --- | --- | --- | --- |
|  | **Total** | **T**ertile 1 | **T**ertile 2 | **T**ertile 3 |  |  | **Total** | **T**ertile 1 | **T**ertile 2 | **T**ertile 3 |  |  |
| **Vitamin C** | | | | | | | | | | | | |
| Age at diagnosis |  |  |  |  | 0.82 | 0.46 |  |  |  |  | 0.31 | 0.54 |
| ≤ 50 | 77/209 | 1.00 (ref.) | 0.38 (0.18, 0.79) | 0.17 (0.06, 0.51) |  |  | 77/209 | 1.00 (ref.) | 0.73 (0.26, 2.01) | 0.24 (0.06, 0.93) |  |  |
| > 50 | 134/351 | 1.00 (ref.) | 0.88 (0.51, 1.52) | 0.47 (0.23, 0.97) |  |  | 134/351 | 1.00 (ref.) | 2.02 (1.04, 3.90) | 1.57 (0.73, 3.39) |  |  |
| FIGO stage |  |  |  |  | 0.56 | 0.84 |  |  |  |  | 0.32 | 0.28 |
| I-II | 48/229 | 1.00 (ref.) | 0.25 (0.08, 0.75) | 0.05 (0.01, 0.23) |  |  | 48/229 | 1.00 (ref.) | 0.54 (0.16, 1.86) | 0.23 (0.04, 1.23) |  |  |
| III-IV | 163/331 | 1.00 (ref.) | 0.74 (0.46, 1.20) | 0.46 (0.25, 0.84) |  |  | 163/331 | 1.00 (ref.) | 1.69 (0.94, 3.02) | 1.12 (0.56, 2.26) |  |  |
| Histological type |  |  |  |  | 0.23 | 0.20 |  |  |  |  | 0.06 | 0.13 |
| Serous | 171/419 | 1.00 (ref.) | 0.76 (0.48, 1.21) | 0.39 (0.21, 0.70) |  |  | 171/419 | 1.00 (ref.) | 1.22 (0.71, 2.08) | 0.71 (0.37, 1.37) |  |  |
| Non-serous | 40/141 | 1.00 (ref.) | 0.26 (0.05, 1.28) | 0.22 (0.02, 1.94) |  |  | 40/141 | 1.00 (ref.) | 1.11 (0.09, 13.38) | 0.50 (0.02, 13.58) |  |  |
| Smoking status |  |  |  |  | 0.10 | 0.05 |  |  |  |  | 0.14 | 0.97 |
| No | 188/501 | 1.00 (ref.) | 0.70 (0.45, 1.10) | 0.37 (0.21, 0.64) |  |  | 186/499 | 1.00 (ref.) | 1.34 (0.79, 2.26) | 0.80 (0.41, 1.54) |  |  |
| Yes | 23/59 | 1.00 (ref.) | 0.01 (0.01, 0.01) | 0.01 (0.01, 0.03) |  |  | 25/61 | 1.00 (ref.) | 0.01 (0.01, 0.01) | 0.01 (0.01, 1.12) |  |  |
| Alcohol drinking |  |  |  |  | 0.58 | 0.34 |  |  |  |  | 0.22 | 0.01 |
| No | 142/372 | 1.00 (ref.) | 0.90 (0.54, 1.50) | 0.43 (0.22, 0.84) |  |  | 176/446 | 1.00 (ref.) | 1.49 (0.84, 2.64) | 0.84 (0.42, 1.67) |  |  |
| Yes | 69/188 | 1.00 (ref.) | 0.21 (0.08, 0.54) | 0.11 (0.04, 0.36) |  |  | 35/114 | 1.00 (ref.) | 1.05 (0.16, 6.82) | 1.76 (0.18, 17.40) |  |  |
| Dietary Supplement |  |  |  |  | 0.56 | 0.83 |  |  |  |  | 0.18 | 0.08 |
| No | 174/449 | 1.00 (ref.) | 0.61 (0.39, 0.96) | 0.33 (0.18, 0.59) |  |  | 145/352 | 1.00 (ref.) | 0.89 (0.47, 1.71) | 0.45 (0.20, 1.02) |  |  |
| Yes | 37/111 | 1.00 (ref.) | 1.49 (0.30, 7.40) | 0.70 (0.10, 5.03) |  |  | 66/208 | 1.00 (ref.) | 1.43 (0.56, 3.70) | 2.30 (0.61, 8.72) |  |  |
| **Retinol** | | | | | | | | | | | | |
| Age at diagnosis |  |  |  |  | 0.01 | 0.11 |  |  |  |  | 0.85 | 0.01 |
| ≤ 50 | 77/209 | 1.00 (ref.) | 1.01 (0.45, 2.30) | 1.00 (0.38, 2.61) |  |  | 77/209 | 1.00 (ref.) | 0.69 (0.23, 2.10) | 1.24 (0.34, 4.53) |  |  |
| > 50 | 134/351 | 1.00 (ref.) | 0.62 (0.36, 1.05) | 0.46 (0.23, 0.91) |  |  | 134/351 | 1.00 (ref.) | 0.86 (0.51, 1.45) | 0.47 (0.19, 1.14) |  |  |
| FIGO stage |  |  |  |  | 0.82 | 0.67 |  |  |  |  | 0.37 | 0.74 |
| I-II | 48/229 | 1.00 (ref.) | 0.58 (0.21, 1.64) | 0.59 (0.16, 2.09) |  |  | 48/229 | 1.00 (ref.) | 0.24 (0.07, 0.83) | 0.19 (0.04, 0.95) |  |  |
| III-IV | 163/331 | 1.00 (ref.) | 0.84 (0.54, 1.32) | 0.71 (0.39, 1.30) |  |  | 163/331 | 1.00 (ref.) | 1.05 (0.63, 1.74) | 1.10 (0.50, 2.45) |  |  |
| Histological type |  |  |  |  | 0.32 | 0.22 |  |  |  |  | 0.70 | 0.72 |
| Serous | 171/419 | 1.00 (ref.) | 0.70 (0.45, 1.08) | 0.75 (0.43, 1.31) |  |  | 171/419 | 1.00 (ref.) | 1.09 (0.67, 1.78) | 0.77 (0.38, 1.56) |  |  |
| Non-serous | 40/141 | 1.00 (ref.) | 1.92 (0.48, 7.63) | 0.10 (0.01, 1.15) |  |  | 40/141 | 1.00 (ref.) | 0.02 (0.01, 0.15) | 0.15 (0.01, 2.10) |  |  |
| Smoking status |  |  |  |  | 0.89 | 0.61 |  |  |  |  | 0.64 | 0.14 |
| No | 188/501 | 1.00 (ref.) | 0.76 (0.50, 1.16) | 0.64 (0.37, 1.11) |  |  | 186/499 | 1.00 (ref.) | 0.92 (0.58, 1.47) | 1.02 (0.50, 2.08) |  |  |
| Yes | 23/59 | 1.00 (ref.) | 0.01 (0.01, 0.01) | 9.46E+25 (1.41E+05, 6.37E+46) |  |  | 25/61 | 1.00 (ref.) | 1.74 (0.08, 38.58) | 0.14 (0.01, 13.36) |  |  |
| Alcohol drinking |  |  |  |  | 0.06 | 0.22 |  |  |  |  | 0.40 | 0.73 |
| No | 142/372 | 1.00 (ref.) | 0.64 (0.39, 1.03) | 0.48 (0.25, 0.91) |  |  | 176/446 | 1.00 (ref.) | 0.98 (0.61, 1.60) | 0.70 (0.33, 1.49) |  |  |
| Yes | 69/188 | 1.00 (ref.) | 2.75 (1.15, 6.61) | 3.32 (1.03, 10.76) |  |  | 35/114 | 1.00 (ref.) | 3.05 (0.66, 14.16) | 6.55 (0.62, 69.14) |  |  |
| Dietary Supplement |  |  |  |  | 0.59 | 0.63 |  |  |  |  | 0.17 | 0.31 |
| No | 174/449 | 1.00 (ref.) | 0.76 (0.49, 1.18) | 0.57 (0.33, 1.01) |  |  | 145/352 | 1.00 (ref.) | 0.82 (0.48, 1.39) | 0.74 (0.34, 1.63) |  |  |
| Yes | 37/111 | 1.00 (ref.) | 0.72 (0.12, 4.28) | 0.70 (0.08, 5.83) |  |  | 66/208 | 1.00 (ref.) | 0.60 (0.19, 1.94) | 0.82 (0.19, 3.57) |  |  |
| **α-carotene** | | | | | | | | | | | | |
| Age at diagnosis |  |  |  |  | 0.35 | 0.38 |  |  |  |  | 0.99 | 0.48 |
| ≤ 50 | 77/209 | 1.00 (ref.) | 1.20 (0.55, 2.63) | 2.03 (0.82, 4.99) |  |  | 77/209 | 1.00 (ref.) | 0.74 (0.36, 1.53) | 0.89 (0.36, 2.21) |  |  |
| > 50 | 134/351 | 1.00 (ref.) | 1.09 (0.65, 1.81) | 1.07 (0.59, 1.95) |  |  | 134/351 | 1.00 (ref.) | 0.91 (0.53, 1.56) | 0.74 (0.38, 1.44) |  |  |
| FIGO stage |  |  |  |  | 0.82 | 0.63 |  |  |  |  | 0.84 | 0.33 |
| I-II | 48/229 | 1.00 (ref.) | 0.20 (0.06, 0.61) | 1.80 (0.56, 5.80) |  |  | 48/229 | 1.00 (ref.) | 0.78 (0.28, 2.14) | 1.11 (0.32, 3.80) |  |  |
| III-IV | 163/331 | 1.00 (ref.) | 1.32 (0.82, 2.12) | 1.04 (0.60, 1.82) |  |  | 163/331 | 1.00 (ref.) | 1.01 (0.63, 1.61) | 0.91 (0.50, 1.63) |  |  |
| Histological type |  |  |  |  | 0.24 | 0.34 |  |  |  |  | 0.78 | 0.39 |
| Serous | 171/419 | 1.00 (ref.) | 1.22 (0.78, 1.90) | 1.30 (0.77, 2.22) |  |  | 171/419 | 1.00 (ref.) | 0.88 (0.56, 1.37) | 0.80 (0.46, 1.39) |  |  |
| Non-serous | 40/141 | 1.00 (ref.) | 0.60 (0.11, 3.35) | 1.10 (0.13, 9.49) |  |  | 40/141 | 1.00 (ref.) | 0.53 (0.10, 2.78) | 0.82 (0.13, 5.26) |  |  |
| Smoking status |  |  |  |  | 0.04 | 0.01 |  |  |  |  | 0.66 | 0.13 |
| No | 188/501 | 1.00 (ref.) | 1.22 (0.79, 1.87) | 1.31 (0.79, 2.17) |  |  | 186/499 | 1.00 (ref.) | 0.97 (0.63, 1.49) | 1.02 (0.60, 1.73) |  |  |
| Yes | 23/59 | 1.00 (ref.) | 0.01 (0.01, 1.90) | 0.01 (0.01, 0.01) |  |  | 25/61 | 1.00 (ref.) | 0.09 (0.01, 0.61) | 0.43 (0.05, 3.62) |  |  |
| Alcohol drinking |  |  |  |  | 0.13 | 0.24 |  |  |  |  | 0.62 | 0.25 |
| No | 142/372 | 1.00 (ref.) | 0.80 (0.47, 1.35) | 0.78 (0.43, 1.44) |  |  | 176/446 | 1.00 (ref.) | 0.90 (0.57, 1.43) | 0.80 (0.46, 1.39) |  |  |
| Yes | 69/188 | 1.00 (ref.) | 3.03 (1.30, 7.07) | 4.07 (1.47, 11.31) |  |  | 35/114 | 1.00 (ref.) | 0.56 (0.12, 2.59) | 1.25 (0.22, 6.96) |  |  |
| Dietary Supplement |  |  |  |  | 0.66 | 0.64 |  |  |  |  | 0.87 | 0.47 |
| No | 174/449 | 1.00 (ref.) | 1.04 (0.67, 1.62) | 1.10 (0.65, 1.89) |  |  | 145/352 | 1.00 (ref.) | 0.77 (0.46, 1.30) | 0.82 (0.43, 1.58) |  |  |
| Yes | 37/111 | 1.00 (ref.) | 0.97 (0.21, 4.39) | 0.47 (0.08, 2.72) |  |  | 66/208 | 1.00 (ref.) | 1.04 (0.49, 2.19) | 0.84 (0.31, 2.23) |  |  |
| **β-carotene** | | | | | | | | | | | | |
| Age at diagnosis |  |  |  |  | 0.93 | 0.44 |  |  |  |  | 0.46 | 0.32 |
| ≤ 50 | 77/209 | 1.00 (ref.) | 0.64 (0.25, 1.62) | 0.72 (0.23, 2.24) |  |  | 77/209 | 1.00 (ref.) | 0.77 (0.34, 1.73) | 0.98 (0.30, 3.25) |  |  |
| > 50 | 134/351 | 1.00 (ref.) | 1.45 (0.85, 2.47) | 1.51 (0.78, 2.90) |  |  | 134/351 | 1.00 (ref.) | 0.62 (0.34, 1.11) | 0.53 (0.26, 1.10) |  |  |
| FIGO stage |  |  |  |  | 0.50 | 0.60 |  |  |  |  | 0.31 | 0.75 |
| I-II | 48/229 | 1.00 (ref.) | 1.18 (0.47, 2.97) | 0.29 (0.07, 1.20) |  |  | 48/229 | 1.00 (ref.) | 0.39 (0.13, 1.16) | 1.73 (0.41, 7.41) |  |  |
| III-IV | 163/331 | 1.00 (ref.) | 1.19 (0.72, 1.98) | 1.25 (0.68, 2.29) |  |  | 163/331 | 1.00 (ref.) | 0.51 (0.31, 0.86) | 0.48 (0.24, 0.93) |  |  |
| Histological type |  |  |  |  | 0.76 | 0.53 |  |  |  |  | 0.56 | 0.94 |
| Serous | 171/419 | 1.00 (ref.) | 1.26 (0.78, 2.03) | 1.07 (0.60, 1.92) |  |  | 171/419 | 1.00 (ref.) | 0.81 (0.49, 1.34) | 0.73 (0.39, 1.35) |  |  |
| Non-serous | 40/141 | 1.00 (ref.) | 1.52 (0.40, 5.73) | 1.16 (0.16, 8.45) |  |  | 40/141 | 1.00 (ref.) | 0.05 (0.01, 0.25) | 0.05 (0.01, 0.56) |  |  |
| Smoking status |  |  |  |  | 0.01 | 0.03 |  |  |  |  | 0.89 | 0.95 |
| No | 188/501 | 1.00 (ref.) | 1.23 (0.78, 1.93) | 1.15 (0.66, 2.02) |  |  | 186/499 | 1.00 (ref.) | 0.57 (0.35, 0.92) | 0.50 (0.27, 0.92) |  |  |
| Yes | 23/59 | 1.00 (ref.) | 0.01 (0.01, -) | 0.01 (0.01, -) |  |  | 25/61 | 1.00 (ref.) | 0.01 (0.01, 0.16) | 0.09 (0.01, 22.68) |  |  |
| Alcohol drinking |  |  |  |  | 0.87 | 0.41 |  |  |  |  | 0.49 | 0.35 |
| No | 142/372 | 1.00 (ref.) | 1.28 (0.77, 2.15) | 1.43 (0.73, 2.79) |  |  | 176/446 | 1.00 (ref.) | 0.55 (0.33, 0.92) | 0.51 (0.26, 0.96) |  |  |
| Yes | 69/188 | 1.00 (ref.) | 1.77 (0.71, 4.41) | 0.85 (0.30, 2.42) |  |  | 35/114 | 1.00 (ref.) | 0.68 (0.15, 3.06) | 0.10 (0.01, 1.06) |  |  |
| Dietary Supplement |  |  |  |  | 0.36 | 0.36 |  |  |  |  | 0.74 | 0.20 |
| No | 174/449 | 1.00 (ref.) | 1.28 (0.80, 2.05) | 0.99 (0.56, 1.78) |  |  | 145/352 | 1.00 (ref.) | 0.57 (0.32, 1.02) | 0.58 (0.28, 1.18) |  |  |
| Yes | 37/111 | 1.00 (ref.) | 1.06 (0.25, 4.50) | 3.87 (0.53, 28.51) |  |  | 66/208 | 1.00 (ref.) | 0.51 (0.23, 1.15) | 0.65 (0.18, 2.29) |  |  |

FIGO, The International Federation of Gynecology and Obstetrics; Ref, reference.

All hazard ratios (95% confidence intervals) were calculated using Cox proportional hazard models.

^1^ Pre-diagnosis model for individual dietary antioxidant nutrients intake adjusted for age at diagnosis, pre-diagnosis BMI, pre-diagnosis total energy intake, pre-diagnosis physical activity, income, education, pre-diagnosis smoking status, pre-diagnosis alcohol drinking, pre-diagnosis dietary supplement, menopause status, parity, histological type, residual lesions, FIGO stage, and other dietary antioxidant nutrients intake before diagnosis.

^2^ Post-diagnosis model for individual dietary antioxidant nutrients intake adjusted for age at diagnosis, post-diagnosis BMI, post-diagnosis total energy intake, post-diagnosis physical activity, income, education, post-diagnosis smoking status, post-diagnosis alcohol drinking, post-diagnosis dietary supplement, menopause status, parity, histological type, residual lesions, FIGO stage, and other dietary antioxidant nutrients intake after diagnosis.

^3^ Indicated *P* value for multiplicative interaction.

^4^ Indicated *P* value for additive interaction.

**Supplementary Table S10. Sensitivity analyses for the associations (hazard ratio and 95% confidence interval) of pre-diagnosis and post-diagnosis** **cumulative and individual dietary antioxidant nutrients intake with overall survival of ovarian cancer**

|  | **Pre-diagnosis** | | | ***P* for**  **trend ^1^** | **Per-SD**  **increase ^2^** | **Post-diagnosis** | | | ***P* for**  **trend ^1^** | **Per-SD increase ^2^** |
| --- | --- | --- | --- | --- | --- | --- | --- | --- | --- | --- |
|  | **Tertile 1** | **Tertile 2** | **Tertile 3** |  |  | **Tertile 1** | **Tertile 2** | **Tertile 3** |  |  |
| **TAC** | | | | | | | | | | |
| 1-y lag before mortality 3 | 1.00 (ref.) | 0.81 (0.56, 1.17) | 0.59 (0.38, 0.92) | 0.02 | 0.76 (0.62, 0.94) | 1.00 (ref.) | 0.80 (0.55, 1.16) | 0.69 (0.44, 1.08) | 0.11 | 0.78 (0.62, 0.99) |
| Energy-adjusted 4 | 1.00 (ref.) | 0.83 (0.60, 1.16) | 0.59 (0.41, 0.83) | 0.01 | 0.80 (0.69, 0.92) | 1.00 (ref.) | 0.85 (0.61, 1.19) | 0.58 (0.41, 0.82) | 0.01 | 0.82 (0.70, 0.95) |
| **CDAI** | | | | | | | | | | |
| 1-y lag before mortality 3 | 1.00 (ref.) | 0.98 (0.66, 1.43) | 0.57 (0.31, 1.05) | 0.06 | 0.74 (0.54, 1.02) | 1.00 (ref.) | 0.85 (0.57, 1.26) | 0.58 (0.32, 1.04) | 0.07 | 0.63 (0.45, 0.89) |
| Energy-adjusted 4 | 1.00 (ref.) | 1.00 (0.71, 1.39) | 0.92 (0.65, 1.30) | 0.62 | 0.87 (0.76, 1.00) | 1.00 (ref.) | 0.85 (0.61, 1.18) | 0.66 (0.47, 0.93) | 0.02 | 0.81 (0.71, 0.94) |
| **Vitamin C** | | | | | | | | | | |
| 1-y lag before mortality 3 | 1.00 (ref.) | 0.65 (0.42, 1.02) | 0.39 (0.22, 0.70) | 0.01 | 0.63 (0.48, 0.81) | 1.00 (ref.) | 1.46 (0.86, 2.47) | 0.82 (0.42, 1.60) | 0.24 | 0.88 (0.67, 1.17) |
| Energy-adjusted 4 | 1.00 (ref.) | 0.84 (0.59, 1.20) | 0.54 (0.36, 0.81) | 0.01 | 0.70 (0.59, 0.84) | 1.00 (ref.) | 1.08 (0.75, 1.57) | 0.84 (0.56, 1.27) | 0.42 | 0.90 (0.75, 1.07) |
| **Retinol** | | | | | | | | | | |
| 1-y lag before mortality 3 | 1.00 (ref.) | 0.73 (0.49, 1.11) | 0.64 (0.37, 1.08) | 0.14 | 1.09 (0.88, 1.35) | 1.00 (ref.) | 0.89 (0.56, 1.43) | 0.65 (0.33, 1.29) | 0.21 | 1.04 (0.83, 1.32) |
| Energy-adjusted 4 | 1.00 (ref.) | 1.10 (0.72, 1.67) | 0.79 (0.49, 1.25) | 0.20 | 1.07 (0.89, 1.29) | 1.00 (ref.) | 1.06 (0.71, 1.59) | 0.97 (0.56, 1.68) | 0.91 | 1.08 (0.89, 1.31) |
| **α-carotene** | | | | | | | | | | |
| 1-y lag before mortality 3 | 1.00 (ref.) | 0.98 (0.64, 1.50) | 1.05 (0.64, 1.73) | 0.78 | 0.93 (0.77, 1.13) | 1.00 (ref.) | 1.17 (0.77, 1.77) | 1.03 (0.60, 1.77) | 0.93 | 0.93 (0.73, 1.19) |
| Energy-adjusted 4 | 1.00 (ref.) | 0.96 (0.66, 1.38) | 1.06 (0.70, 1.61) | 0.70 | 0.95 (0.81, 1.11) | 1.00 (ref.) | 1.00 (0.69, 1.46) | 0.92 (0.59, 1.44) | 0.70 | 0.95 (0.78, 1.17) |
| **β-carotene** | | | | | | | | | | |
| 1-y lag before mortality 3 | 1.00 (ref.) | 1.22 (0.78, 1.90) | 1.20 (0.69, 2.11) | 0.63 | 0.89 (0.71, 1.12) | 1.00 (ref.) | 0.47 (0.29, 0.76) | 0.52 (0.29, 0.93) | 0.09 | 0.76 (0.58, 0.99) |
| Energy-adjusted 4 | 1.00 (ref.) | 1.18 (0.81, 1.73) | 1.00 (0.65, 1.53) | 0.82 | 0.88 (0.74, 1.06) | 1.00 (ref.) | 0.70 (0.48, 1.03) | 0.53 (0.33, 0.84) | 0.01 | 0.79 (0.65, 0.97) |

CDAI, composite dietary antioxidant index; Ref, reference; TAC, total antioxidant capacity.

All hazard ratios (95% confidence intervals) were calculated using Cox proportional hazard models.

^1^ *P* for trend was tested by assigning medians of the tertiles for TAC and CDAI in all patients.

^2^ Percentage change in ovarian cancer survival for each standard deviation increase in TAC and CDAI.

^3^ Excluded patients with a survival duration shorter than one year.

^4^ Adjusted for total energy intake using the residual method.

**Supplementary Table S11. E-values for patients with ovarian cancer according to pre-diagnosis and post-diagnosis cumulative and individual dietary antioxidant nutrients intake**

|  | **Pre-diagnosis** | | | **Post-diagnosis** | | |
| --- | --- | --- | --- | --- | --- | --- |
|  | **Tertile 1** | **Tertile 2** | **Tertile 3** | **Tertile 1** | **Tertile 2** | **Tertile 3** |
| **TAC** | | | | | | |
| HR | 1.00 (ref.) | 0.85 | 0.58 | 1.00 (ref.) | 0.65 | 0.57 |
| E-values * | - | - | 2.27 | - | - | 2.31 |
| **CDAI** | | | | | | |
| HR | 1.00 (ref.) | 1.09 | 0.7 | 1.00 (ref.) | 0.76 | 0.57 |
| E-values * | - | - | 1.88 | - | - | 2.31 |
| **Vitamin C** | | | | | | |
| HR | 1.00 (ref.) | 0.68 | 0.36 | 1.00 (ref.) | 1.25 | 0.81 |
| E-values * | - | - | 3.44 | - | - | 1.58 |
| **Retinol** | | | | | | |
| HR | 1.00 (ref.) | 0.78 | 0.65 | 1.00 (ref.) | 0.88 | 0.89 |
| E-values * | - | - | 2.03 | - | - | 1.39 |
| **α-carotene** | | | | | | |
| HR | 1.00 (ref.) | 1.07 | 1.18 | 1.00 (ref.) | 0.97 | 0.95 |
| E-values * | - | - | 1.49 | - | - | 1.23 |
| **β-carotene** | | | | | | |
| HR | 1.00 (ref.) | 1.18 | 1.12 | 1.00 (ref.) | 0.56 | 0.55 |
| E-values * | - | - | 1.38 | - | - | 2.39 |

CDAI, composite dietary antioxidant index; HR, hazard ratio; Ref, reference; TAC, total antioxidant capacity.

* RR ≈ (1 - 0.5^sqrt(HR)^)/(1 - 0.5^sqrt(1/HR)^); RR<1: E-value = 1/RR + sqrt[1/RR × (1/RR - 1)]; RR>1: E-value = RR + sqrt[RR × (RR - 1)].
